# High-intensity interval training and continuous glucose monitoring-derived glycemic outcomes in adults with type 2 diabetes: a systematic review and meta-analysis

**DOI:** 10.3389/fendo.2026.1834479

**Published:** 2026-06-17

**Authors:** Jun Li, Jin Yuan, Yong Zhang, Quanwen Zeng, Haifeng Ma

**Affiliations:** 1 School of Athletic Performance, Shanghai University of Sport, Shanghai, China; 2 Faculty of Sports Science, Ningbo University, Ningbo, China; 3 School of Physical Education, Anhui Polytechnic University, Wuhu, China

**Keywords:** 24-hour mean glucose, continuous glucose monitoring, high-intensity interval training, meta-analysis, postprandial glucose, systematic review, time spent in hyperglycemia, type 2 diabetes

## Abstract

**Background:**

Type 2 diabetes (T2D) is characterized by chronic hyperglycemia and marked within-day glucose fluctuations. Compared with conventional glycemic markers, continuous glucose monitoring (CGM)-derived metrics provide complementary information on overall glycemic exposure, postprandial responses, and glycemic variability. Although high-intensity interval training (HIIT) has shown promise for improving glycemic control in T2D, its effects on CGM-derived outcomes have not been systematically synthesized.

**Objective:**

To systematically review and meta-analyze the effects of HIIT on CGM-derived glycemic outcomes in adults with T2D, compared with non-exercise control (CON) or other exercise interventions.

**Methods:**

PubMed, Embase, Web of Science, the Cochrane Library, and EBSCOhost were searched from inception to January 26, 2026. Interventional studies comparing HIIT with CON or other exercise interventions and reporting CGM-derived outcomes were included. When data were suitable for quantitative pooling, pooled estimates were calculated from change-from-baseline values using fixed- or random-effects models. Risk of bias was assessed using RoB 2, RoB 2 for crossover trials, or ROBINS-I, and certainty of evidence was evaluated with GRADE.

**Results:**

Twelve studies involving 177 participants were included. Compared with CON, HIIT reduced 24-hour mean glucose, postprandial glucose, and time spent in hyperglycemia, while its effect on mean amplitude of glycemic excursions (MAGE) remained uncertain. Compared with moderate-intensity continuous training (MICT), HIIT further reduced 24-hour mean glucose and time spent in hyperglycemia, but no significant between-group difference was found for MAGE. Overall certainty of evidence ranged from very low to low.

**Conclusions:**

HIIT may improve selected CGM-derived glycemic outcomes in adults with T2D, particularly postprandial glucose and hyperglycemic exposure. Compared with MICT, HIIT may offer additional benefits for reducing 24-hour mean glucose and hyperglycemic time. However, confidence in these findings is limited by the small number of studies, methodological limitations, and heterogeneity.

## 1 Introduction

Management of type 2 diabetes (T2D) increasingly considers not only long-term average glycemia but also within-day glycemic exposure, postprandial glucose responses, and glucose fluctuation patterns. In this context, continuous glucose monitoring (CGM) has become an important tool for assessing short-term and day-to-day glycemic responses in adults with T2D, because it provides frequent, near-continuous measurements of interstitial glucose and enables a more detailed characterization of glycemic profiles through metrics such as 24-hour mean glucose, time in range (TIR), time above range (TAR), time below range (TBR), and other glycemic variability (GV)-related indices (1–3).

Conventional glycemic markers, including fasting blood glucose and glycated hemoglobin (HbA1c), remain clinically important in T2D management, but they primarily reflect blood glucose at a single time point or average glycemic exposure over a longer period. By contrast, CGM reflects changes in interstitial glucose and can provide complementary information on within-day glycemic exposure, postprandial excursions, and glucose fluctuations (1–3). CGM should therefore not be regarded as a simple replacement for conventional glycemic markers, but rather as an additional approach that captures complementary information on glycemic control through interstitial glucose dynamics. Accumulating evidence further suggests that GV may reflect the stability of glycemic control and may also be associated with hypoglycemia risk and the development and progression of diabetes-related complications (4–6). Accordingly, CGM-derived glycemic outcomes are of particular interest in exercise intervention research.

Regular exercise is a cornerstone of T2D management. Current consensus statements generally recommend that adults with T2D accumulate 150–300 min of moderate-intensity aerobic physical activity per week, 75–150 min of vigorous-intensity aerobic activity, or an equivalent combination of both, together with resistance training and reductions in sedentary behavior (7). However, in real-world settings, long-term adherence to recommended exercise volumes remains suboptimal, and lack of time is consistently reported as an important barrier (7, 8). High-intensity interval training (HIIT), characterized by repeated bouts of vigorous exercise interspersed with recovery periods, has therefore attracted increasing attention as a time-efficient alternative or complement to traditional continuous aerobic training (7, 9). At the same time, the feasibility and safety of HIIT should not be assumed to be uniform across all individuals with chronic disease. Long-term adherence, tolerability, and the ability to consistently achieve target intensity may depend on disease severity, supervision, program design, and individual characteristics (10, 11). Previous systematic reviews and meta-analyzes have generally suggested that HIIT may improve selected glycemic, cardiorespiratory, and cardiometabolic outcomes in adults with T2D. However, the magnitude and consistency of these effects have not been uniformed across reviews, partly because of differences in participant characteristics, training modality, exercise dose, work-to-recovery structure, comparator conditions, and outcome definitions (9, 12–14).

Although CGM offers a higher-resolution approach for evaluating short-term glycemic responses to exercise interventions, CGM-derived outcomes, as well as their definitions, calculations, and reporting formats, remain incompletely standardized across studies (1, 15). International consensus statements have provided an interpretive framework for core CGM metrics such as TIR, TAR, and TBR, but variation persists in the selection and presentation of 24-hour mean glucose, postprandial glucose, mean amplitude of glycemic excursions (MAGE), and other GV-related indices (1, 15). In addition, although CGM has become increasingly relevant in both research and clinical practice, its real-world application may still be influenced by device availability, wear-time compliance, analytical consistency, and differences in reporting practices. As a result, the effects of HIIT on CGM-derived glycemic outcomes in adults with T2D, as well as the certainty of the available evidence, have not yet been comprehensively synthesized.

Therefore, the present study systematically reviewed interventional evidence on HIIT in adults with T2D, with a specific focus on CGM-derived glycemic outcomes in comparison with non-exercise control conditions or other structured exercise interventions. Where methodologically appropriate, meta-analyzes and evidence grading were further conducted to help inform CGM-informed and individualized exercise prescription in adults with T2D.

## 2 Methods

This systematic review and meta-analysis was conducted in accordance with the Cochrane Handbook for Systematic Reviews of Interventions (16) and reported in accordance with the Preferred Reporting Items for Systematic Reviews and Meta-Analyzes 2020 (PRISMA 2020) statement (17). The review protocol was prospectively registered in PROSPERO (CRD42024574204), and the registration record was checked and updated during revision to reflect the current stage of the review.

### 2.1 Search strategy and study selection

Two reviewers (Jun Li and Jin Yuan) systematically searched PubMed, Embase, Web of Science, the Cochrane Library, and EBSCOhost (SPORTDiscus) from database inception to January 26, 2026. Only peer-reviewed original studies published in English were considered eligible. The search strategy combined controlled vocabulary and free-text terms. Using PubMed MeSH terms as the foundation, the search was structured around three core concepts: “Diabetes Mellitus, Type 2,” “High-Intensity Interval Training,” and “Continuous Glucose Monitoring.” Relevant synonyms, abbreviations, and common variants were incorporated to maximize sensitivity. Terms related to type 2 diabetes included, but were not limited to, T2D and T2DM. Terms related to continuous glucose monitoring included CGM. Because terminology for high-intensity interval exercise remains partly inconsistent across studies, the HIIT-related search terms were expanded to include HIIT, HIT, HIIE, LVHIIT, HVHIIT, REHIT/REHIIT, SIT, interval training, and interval aerobic training. The full search strategies for all databases and the detailed search process are provided in **Supplementary Table 1**.

During manuscript revision, an additional update search was conducted across the originally searched databases to assess whether any eligible studies had been missed after the initial screening. The supplementary search terms included “type II diabetes,” “real-time glucose monitoring,” “flash glucose monitoring,” “aerobic interval training,” and “interval exercise.” No additional eligible controlled HIIT intervention studies published in 2024, 2025, or January 2026 were identified after applying the prespecified PICOS criteria.

All retrieved records were imported into EndNote X9 for deduplication. Subsequently, two reviewers independently conducted title and abstract screening, followed by full-text assessment of potentially eligible studies. Any disagreements were resolved through discussion and consensus; when consensus could not be reached, a third reviewer (Haifeng Ma) acted as an adjudicator. In addition, the reference lists of included studies were manually screened to identify any further relevant studies that might have been missed in the database searches. The study selection process was documented using a PRISMA flow diagram.

### 2.2 Eligibility criteria

The inclusion and exclusion criteria were established according to the PICOS framework.

#### 2.2.1 Inclusion criteria

Studies were considered eligible if they met the following criteria:

Participants (P):

Adults aged ≥18 years with T2D.

Intervention (I):

Structured HIIT.

Comparator (C):

A non-exercise control (CON) or another structured exercise intervention, such as moderate-intensity continuous training (MICT), resistance exercise, or combined aerobic-resistance exercise, provided that the independent effect of HIIT could be isolated.

Outcomes (O):

Studies were required to report at least one CGM-derived glycemic outcome. Any type of CGM system was eligible, including real-time CGM and intermittently scanned CGM. Eligible outcomes included, but were not limited to:

1. 24-hour mean glucose**;**
2. postprandial glucose (PPG), defined either as mean glucose values directly reported for a specified postprandial time window or as mean values derived from the reported area under the curve (AUC) and the corresponding time window;
3. GV metrics, such as mean amplitude of glycemic excursions (MAGE);
4. TAR and/or TBR, reported in minutes or convertible to minutes. To improve comparability across studies, priority was given to thresholds commonly used in international consensus statements, namely hyperglycemia >10.0 mmol/L and hypoglycemia<3.9 mmol/L. Studies using different thresholds were retained for narrative synthesis. They were considered for quantitative synthesis or sensitivity analysis only when the threshold was clinically comparable to the consensus threshold, the reported unit could be harmonized to minutes, the direction of interpretation was consistent with the primary outcome, and sufficient effect-size and variance information was available. Studies using substantially different thresholds, unclear analytical windows, or insufficient variance data were not pooled.

Study design (S):

Interventional controlled trials, including randomized and non-randomized studies, with either parallel-group or crossover designs.

#### 2.2.2 Exclusion criteria

Studies were excluded if they met any of the following criteria:

1. ineligible publication type, including reviews, systematic reviews, meta-analyzes, observational studies, case reports or case series, conference abstracts, study protocols, trial registrations without results, dissertations, or other gray literature;
2. ineligible or non-separable population, including individuals with type 1 diabetes, gestational diabetes mellitus, children or adolescents, or populations primarily characterized by severe diabetes-related complications or other special clinical conditions. For studies including mixed diabetes populations, data were eligible only when results for adults with T2D were reported separately or could be reasonably obtained. If T2D-specific data could not be separated from type 1 diabetes, gestational diabetes, or other populations, the study was excluded from quantitative synthesis and, where relevant, summarized narratively only;
3. ineligible intervention, including studies in which exercise was not the main intervention, or in which HIIT was combined with medication, dietary intervention, or other exercise modalities such that the independent effect of HIIT could not be separated;
4. unavailable or unusable quantitative data, including studies for which the full text or essential outcome data could not be obtained, duplicate or overlapping reports without separable data, or studies in which key outcome data could not support a valid effect estimate and corresponding variance for the planned synthesis. Unusable data included missing or non-derivable variance estimates, non-convertible units, unclear or incompatible outcome windows, and crossover-trial summaries that lacked paired effect estimates, change scores, within-person variance estimates, or reliable first-period data. Studies with unusable quantitative data were not automatically excluded from the review; when relevant, they were retained for narrative synthesis.

### 2.3 Data extraction

During revision, all extracted outcome data were rechecked against the original articles and **Supplementary Materials**, with particular attention to crossover trials and to how outcome data were summarized and reported. Data extraction was independently performed by two reviewers (Jun Li and Jin Yuan) and cross-checked for accuracy. Any disagreements were resolved through discussion, and when necessary, a third reviewer (Haifeng Ma) acted as an adjudicator.

The following information was extracted from each included study:

1. general study characteristics, including first author, year of publication, study design, and comparator type;
2. sample characteristics, including sample size, age, sex, and body mass index (BMI);
3. CGM-related information, including CGM type, sampling interval, monitoring duration, timing of CGM assessment, and any control requirements for medication, diet, and physical activity during the monitoring period;
4. medication-related information where available, including glucose-lowering medication, antihypertensive medication, beta-blocker use, and whether medication use was reported or controlled during the intervention and CGM monitoring periods;
5. exercise intervention characteristics, including exercise modality, intensity indicators, training frequency, session duration, intervention period, and whether HR-based targets were derived from exercise testing under usual medication conditions;
6. complementary intensity indicators, such as RPE, workload, power output, speed/grade, or oxygen uptake, when reported;
7. outcome data, including means and standard deviations for CGM-derived glycemic outcomes.

The main CGM-derived outcomes extracted for this review included 24-hour mean glucose, PPG, GV metrics such as MAGE, TAR, and TBR.

To improve comparability across studies, several prespecified data conversions and harmonization procedures were applied:

1. glucose values were standardized to mmol/L; when reported in mg/dL, values were converted using mg/dL ÷ 18;
2. when TAR or TBR was reported as a percentage, it was converted to minutes using percentage × total monitoring duration;
3. for PPG, directly reported mean glucose values for a specified postprandial time window were preferentially extracted; if postprandial outcomes were reported as AUC, the corresponding mean glucose value for that time window was calculated as AUC ÷ time window;
4. when 24-hour AUC was reported, it was converted to 24-hour mean glucose using AUC ÷ 24 h;
5. when continuous data were reported as median (interquartile range) or mean (range), established statistical methods were used, where feasible, to estimate mean ± standard deviation, and the conversion procedures were described in the **Supplementary Material**.

For TAR and TBR, priority was given to the thresholds recommended by international consensus statements, namely hyperglycemia >10.0 mmol/L and hypoglycemia<3.9 mmol/L. Studies using similar but slightly different thresholds (e.g., hyperglycemia defined as >9.0 mmol/L or hypoglycemia defined as<4.0 mmol/L) were extracted and described in the narrative synthesis. They were entered into sensitivity analyzes only when the threshold difference was judged to be clinically close to the main definition, the outcome direction remained comparable, the unit could be harmonized, and sufficient effect-size and variance information was available.

For crossover trials, paired or within-person effect estimates were prioritized whenever these were explicitly reported or could be reliably derived. Specifically, paired differences and their corresponding variance estimates or standard deviations were preferentially extracted because these best reflect the within-participant nature of crossover comparisons. If appropriate paired data were unavailable, data from the first study period were considered as a conservative option, where feasible, to reduce the potential influence of carryover effects and avoid the inappropriate reuse of repeated observations from the same participants in quantitative synthesis. In cases where neither appropriate paired estimates nor reliable first-period data were available, a conservative approach was adopted: such studies were retained in the narrative synthesis only and were not included in the quantitative meta-analysis. This strategy was implemented to avoid treating non-paired summary data from crossover trials as though they were derived from independent parallel-group comparisons, to prevent unsupported assumptions about within-person correlation, and to avoid double-counting participant data. For studies with missing key quantitative data, particularly crossover trials lacking paired effect estimates or summary data for change values, the corresponding authors were contacted where feasible to request additional information. If the necessary data could still not be obtained, the study was handled according to the prespecified rules described above.

For Marcotte-Chénard et al. (25), extractable CGM metrics were identified from **Supplementary Table 2** of the original publication. The reported mean ± SD values were reviewed against the prespecified outcome definitions and data requirements of the present review. When the available data provided methodologically appropriate effect estimates and variance information, they were included in the quantitative synthesis. When such information was insufficient, the corresponding findings were summarized narratively rather than pooled. If neither appropriate paired effect estimates nor first-period data could be obtained, a conservative approach was adopted: the study was included only in the narrative synthesis and not in the quantitative meta-analysis. This strategy aimed to prevent misinterpretation of non-paired summary data from crossover trials as if they were derived from independent parallel-group comparisons, to avoid making unsupported assumptions about within-person correlation, and to prevent the double-counting of participant data. For studies with missing key quantitative data, especially in crossover trials where paired effect estimates or summary change data were lacking, the corresponding authors were contacted when possible to request additional information; if the necessary data could not be obtained, the study was processed according to the predefined rules outlined above. For multi-arm parallel trials, where multiple HIIT intervention arms shared a common control group, the shared control group sample size was proportionally split across comparisons as recommended by the Cochrane Handbook to avoid unit-of-analysis errors and improper weighting of the control group.

### 2.4 Risk of bias assessment and certainty of evidence

The risk of bias of the included studies was independently assessed by two reviewers (Jun Li and Jin Yuan), and the results were cross-checked for consistency. Any disagreements were resolved through discussion, and, when necessary, a third reviewer (Haifeng Ma) acted as an adjudicator. Because the included evidence comprised randomized parallel trials, randomized crossover trials, and non-randomized interventional studies, risk-of-bias tools were selected according to study design. The Cochrane Risk of Bias 2 (RoB 2) tool was used for randomized parallel trials, the RoB 2 tool for randomized crossover trials was used for randomized crossover studies, and ROBINS-I was used for non-randomized interventional studies. These tools provide domain-based, outcome-level, and design-sensitive assessments of bias, covering domains such as the randomization process, deviations from intended interventions, missing outcome data, measurement of the outcome, selection of the reported result, confounding in non-randomized studies, and period or carryover effects in crossover trials. This approach is consistent with contemporary Cochrane guidance for intervention reviews (16, 18, 19). Given the inclusion of different study designs, a single summary quality-score approach was not used; instead, domain-based risk-of-bias tools were selected according to study design.

For randomized parallel trials, the RoB 2 tool was used to assess risk of bias across the following domains: the randomization process, deviations from intended interventions, missing outcome data, measurement of the outcome, and selection of the reported result. The overall risk of bias for each outcome was judged as “low risk,” “some concerns,” or “high risk.” For randomized crossover trials, the RoB 2 tool for randomized crossover trials was applied. In addition to the domains above, particular attention was paid to design-specific sources of bias, including carryover effects, period effects, and whether the statistical analysis appropriately accounted for the paired structure of the data. When crossover-specific sources of bias were not adequately addressed, this was reflected in the overall risk-of-bias judgment.

For non-randomized interventional studies, the ROBINS-I (Risk Of Bias In Non-randomized Studies - of Interventions) tool was used. The following domains were evaluated: bias due to confounding, bias in the selection of participants, bias in the classification of interventions, bias due to deviations from intended interventions, bias due to missing data, bias in the measurement of outcomes, and bias in the selection of the reported result. The overall risk of bias was judged as “low,” “moderate,” “serious,” “critical,” or “no information.”

Risk-of-bias assessments were presented separately according to study design and interpreted in light of the methodological characteristics of each design. For outcomes included in the meta-analysis, sensitivity analyzes were conducted when the prespecified feasibility criteria described in the Statistical analysis section were met, in order to examine the potential influence of risk of bias on the robustness of the pooled estimates.

The certainty of evidence for meta-analyzed outcomes was evaluated using the GRADE (Grading of Recommendations Assessment, Development and Evaluation) approach across five domains: risk of bias, inconsistency, indirectness, imprecision, and publication bias. The certainty of evidence was rated as high, moderate, low, or very low (20). For evidence derived from non-randomized interventional studies, limitations related to study design were further considered in the certainty assessment. Where there was substantial clinical heterogeneity, variation in outcome definitions, or insufficient sample size, the certainty of evidence was downgraded in accordance with GRADE principles.

### 2.5 Statistical analysis

Statistical analyzes were performed using Review Manager (RevMan), version 5.3. Pooled effect estimates were calculated using the inverse-variance method. For continuous outcomes measured on the same scale, results were expressed as mean differences (MDs) with corresponding 95% confidence intervals (95% CIs).

This review prioritized the extraction and pooling of change-from-baseline values. For parallel-group trials, the between-group effect was defined as the difference between the change in the HIIT group and the change in the comparator group. When change scores were not directly reported, they were derived from baseline and post-intervention values where feasible. When the standard deviation of the change score (SD_change) was not reported, it was estimated using the formula recommended in the Cochrane Handbook (16):


$$SD_{change}=\sqrt{SD_{baseline}^{2}+SD_{final}^{2}-2r\times SD_{baseline}\times SD_{final}}$$

In the main analysis, a correlation coefficient of r = 0.50 was assumed, and sensitivity analyzes were conducted using r = 0.25 and r = 0.75 to assess the robustness of the combined results under different correlation coefficient assumptions. If change values or their variance were neither directly reported nor reliably derivable, the study was handled according to the crossover trial rules, or was included only in the narrative synthesis and not in the quantitative meta-analysis.

For crossover trials, only those studies that explicitly reported paired effect estimates or could reliably derive them were included in the quantitative synthesis; otherwise, they were treated as descriptive analysis. When both parallel trials and crossover trials were included for the same outcome, quantitative synthesis was limited to effect estimates that were methodologically suitable for merging. If non-paired summary data from crossover trials could substantively impact the validity of the combined effect, they were not treated as independent parallel-group data. For crossover trials lacking paired effect estimates, this study did not apply a uniform assumption of correlation, as doing so could introduce additional uncertainty and substantially affect the combined effect estimates. Although analyzing only first-phase data is a conservative approach, this method was not always feasible in this study because some included studies did not adequately report first-phase outcome data.

Between-study heterogeneity was assessed using Cochran’s Q test and the I^2^ statistic. A random-effects model was used when I^2^ > 50%; otherwise, a fixed-effect model was applied.

Sensitivity analyzes were performed only when the available data were sufficient to support a meaningful robustness check. Leave-one-out analyzes were performed when at least three studies or comparison units were available for an outcome. Sensitivity analyzes based on alternative correlation assumptions for SD_change were performed when change-score standard deviations had to be imputed. Threshold-based sensitivity analyzes for TAR or TBR were performed only when alternative thresholds were clinically comparable and quantitatively convertible. Sensitivity analyzes were not conducted when an outcome was summarized narratively only, when fewer than three studies or comparison units were available, or when data were too sparse or too heterogeneous to support a meaningful robustness check.

Publication bias was assessed qualitatively using funnel plots only when at least 10 studies or comparison units were available for a given outcome. This threshold was not arbitrary; it was selected according to standard methodological guidance (16, 21) because funnel plot asymmetry is difficult to interpret and has low power in small meta-analyzes. Therefore, funnel plots were treated as exploratory visual tools and were not interpreted as definitive evidence of publication bias. Subgroup analyzes were performed only when all of the following criteria were met: I^2^ > 50%, at least 10 studies or comparison units were available for a given outcome, and each subgroup contained at least two studies or comparison units. Prespecified subgroup variables included age, sex, BMI, intervention duration, and CGM monitoring window/time point (e.g., within 48 h after exercise *vs*. beyond 48 h). All statistical tests were two-sided, and p< 0.05 was considered statistically significant.

## 3 Results

Meta-analyzes were conducted using only outcome data that provided effect estimates and variance information suitable for quantitative pooling. For crossover trials, paired effect estimates or other methodologically appropriate variance estimates were prioritized when available. For Marcotte-Chénard et al. (25), extractable CGM data from the **Supplementary Materials** were assessed separately for each outcome and comparison. Data that met the prespecified requirements for quantitative synthesis were included in the relevant pooled analyzes, whereas outcomes or comparisons without suitable effect estimates or variance information were summarized narratively.

### 3.1 Study selection

**Figure 1:**
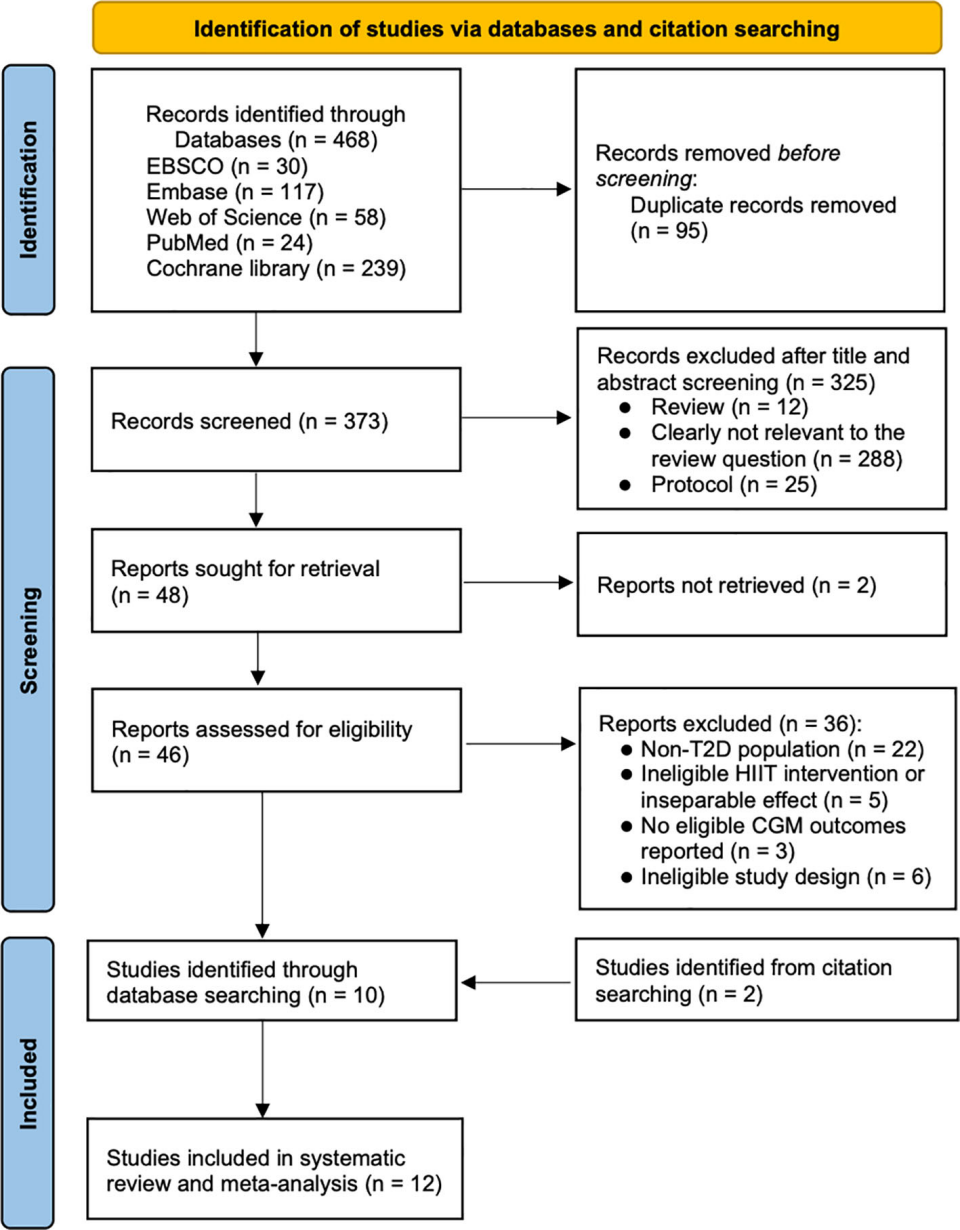
Flow diagram of the literature search and study selection.

A total of 468 records were identified through searches of the five databases. After deduplication in EndNote X9, 373 records remained for title and abstract screening, of which 325 were excluded. Forty-eight reports were then assessed for eligibility. At the full-text stage, 36 reports were excluded because they did not meet the prespecified population, intervention, comparator, outcome, design, or data-availability criteria. Two additional studies were identified through backward screening of reference lists. Ultimately, 12 studies were included in this systematic review and meta-analysis (**Figure 1**). Although the literature search covered records from database inception to January 26, 2026, the eligible included studies were published between 2011 and 2023. This time span should be considered when interpreting the findings, particularly because CGM technology, data processing procedures, and reporting practices evolved substantially during this period.

### 3.2 Risk of bias assessment

Risk of bias was assessed according to study design using RoB 2 for randomized parallel trials, RoB 2 for crossover trials for crossover studies, and ROBINS-I for the non-randomized interventional study. Of the included studies, 3 were randomized parallel trials, 8 were crossover trials, and 1 was a non-randomized interventional study. Detailed risk-of-bias assessments, stratified by study design, are presented in the **Supplementary Figure 1**, with randomized parallel trials, crossover trials, and non-randomized interventional studies shown in **Supplementary Figures S1A–S2C**.

Among the randomized parallel trials, two studies were judged as having some concerns, and one study was judged as high risk. Among the crossover trials, six studies were judged as having some concerns, and two studies were judged as high risk. The only non-randomized interventional study was judged as having serious risk of bias.

Overall, the main methodological limitations of the included studies were related to insufficient information on the randomization process, inadequate control of period and carryover effects in crossover designs, deviations from intended interventions, and missing outcome data. Therefore, the findings of the subsequent meta-analyzes should be interpreted cautiously in light of both the risk of bias and the certainty of evidence.

### 3.3 Study characteristics

#### 3.3.1 Participant characteristics

The 12 included studies comprised three randomized parallel trials, eight crossover trials, and one non-randomized interventional study. Sample sizes were small, ranging from 7 to 32 participants, and the total sample included 177 adults with T2D. Participants were generally middle-aged to older adults and were mostly classified as overweight or obese according to reported BMI values. Detailed participant characteristics are presented in **Table 1**.

**Table 1:** Study and participant characteristics.

Study	Year	Study design	Comparator	Total (n)	Sex (M/F)	Age (years)	BMI (kg/m^2^)	Main CGM outcomes
Little et al. (34)	2011	Non-randomized interventional study	Pre-post HIIT	8	NR	62.5 ± 7.6	31.7 ± 5.8	24-hour mean glucose
Gillen et al. (44)	2012	Crossover trial	HIIT *vs* CON	7	NR	62.0 ± 3.0	30.5 ± 1.9	PPG, time in hyperglycemia
Karstoft et al. (45)	2013	Randomized parallel trial	HIIT *vs* MICT *vs* CON	32	20/12	58-61 (group means)	29.0-29.9 (group means)	Mean glucose, time in hyperglycemia
Karstoft et al. (46)	2014	Crossover trial	HIIT *vs* MICT *vs* CON	10	7/3	60.3 ± 2.3	28.3 ± 1.1	Mean glucose, time in hyperglycemia
Terada et al. (22)	2016	Crossover trial	HIIT/MICT/CON (fed or fasted conditions)	10	2/8	60.0 ± 6.0	30.8 ± 5.4	Mean glucose, GV, time in hyperglycemia
Ruffino et al. (47)	2017	Crossover trial	REHIT *vs* walking	16	16/0	55.0 ± 5.0	30.6 ± 2.8	Mean glucose
Karstoft et al. (26)	2017	Crossover trial	HIIT *vs* MICT *vs* CON	14	11/3	65.0 ± 2.0	NR	Mean glucose, TAR/TBR, MAGE
Winding et al. (27)	2018	Randomized parallel trial	HIIT *vs* MICT *vs* CON	32	19/13	54-58 (group means)	27.4-28.1 (group means)	Mean glucose and other CGM-derived glycemic outcomes
Metcalfe et al. (24)	2018	Crossover trial	HIIT *vs* MICT *vs* REHIT *vs* CON	11	11/0	52.0 ± 6.0	29.7 ± 3.1	24-hour mean glucose, PPG, MAGE
Savikj et al. (23)	2019	Crossover trial	Morning HIIT *vs* afternoon HIIT	11	11/0	60.0 ± 2.0	27.5 ± 0.6	24-hour mean glucose
Chénard et al. (28)	2021	Randomized parallel trial	HIIT *vs* MICT	12	0/12	65.6 ± 4.2	37.2 ± 9.2	Mean glucose, TAR/TBR, MAGE
Chénard et al. (25)	2023	Crossover trial	HIIT1 *vs* HIIT4 *vs* CON	14	0/14	69.9 ± 4.3	33.2 ± 5.6	Mean glucose, GV, time in hyperglycemia

HIIT, high-intensity interval training; MICT, moderate-intensity continuous training; CON, non-exercise control; CGM, continuous glucose monitoring; GV, glycemic variability; TAR, time above range; TBR, time below range; MAGE, mean amplitude of glycemic excursions; PPG, postprandial glucose; NR, not reported.

#### 3.3.2 CGM characteristics

CGM protocols varied across studies in device type, wear duration, sampling interval, timing of assessment, and analytical window. Although most studies recorded or controlled at least one contextual factor, such as medication use, diet, or physical activity during monitoring, the level of detail was inconsistent. Information on CGM devices and monitoring protocols is summarized in **Supplementary Table 2**.

#### 3.3.3 Exercise intervention characteristics

HIIT protocols also varied substantially. The main formats included short-interval cycling protocols, interval walking protocols, reduced-exertion or sprint-based protocols, and 4 × 4-min interval protocols. Interval-walking studies typically used alternating faster and slower walking bouts; intensity anchors were reported in study-specific terms, such as VO_2_peak, peak energy-expenditure rate, or HRmax. Because walking speed, incline, testing modality, and intensity anchoring differed across studies, these protocols should be interpreted as study-defined vigorous or high-intensity interval walking rather than directly comparable conventional walking speeds. Many studies used HR-based monitoring, and some also reported RPE or workload; however, the handling of beta-blocker use and medication-related HR attenuation was not consistently described. Exercise prescription details are provided in **Supplementary Table 3**.

### 3.4 CGM-derived outcomes

After re-evaluating the handling of crossover trials, the affected pooled analyzes were rerun using only studies with methodologically appropriate quantitative data, whereas studies without valid paired effect estimates were retained for narrative synthesis only.

#### 3.4.1 24-hour mean glucose

**Figure 2:**
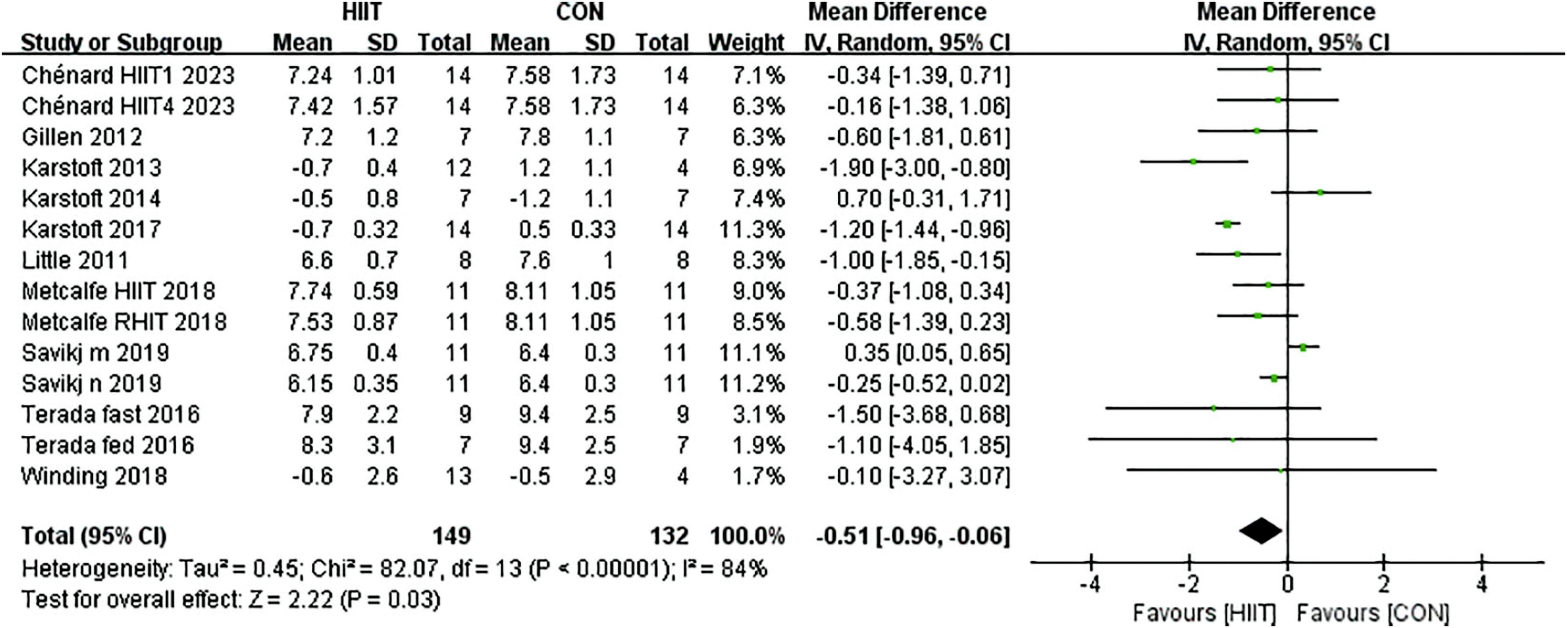
Effect of HIIT versus CON on mean 24-h glucose.

For the HIIT *vs*. CON comparison, 14 comparison units were included in the meta-analysis. Because the heterogeneity between studies was substantial (Chi^2^ = 82.07, p< 0.0001; I^2^ = 84%), a random-effects model was applied. The pooled results showed that, compared with non-exercise control, HIIT was associated with a reduction in 24-hour mean glucose (MD = −0.51 mmol/L, 95% CI −0.96 to −0.06, p = 0.03; **Figure 2**), although the result should be interpreted with caution due to the substantial heterogeneity and the sensitivity to individual studies. Leave-one-out sensitivity analyzes indicated that the heterogeneity remained generally high after exclusion of individual comparison units. However, the statistical significance of the pooled effect changed in some cases after the exclusion of Karstoft 2013, Karstoft 2017, or Little 2011, suggesting that the result has limited robustness. As at least 10 comparison units were available for this outcome, a funnel plot was generated according to the prespecified criteria, suggesting the possibility of small-study effects or potential publication bias (**Supplementary Figure 2A**). According to the GRADE assessment, the certainty of evidence for this outcome was very low (**Supplementary Table 4**).

**Figure 3:**
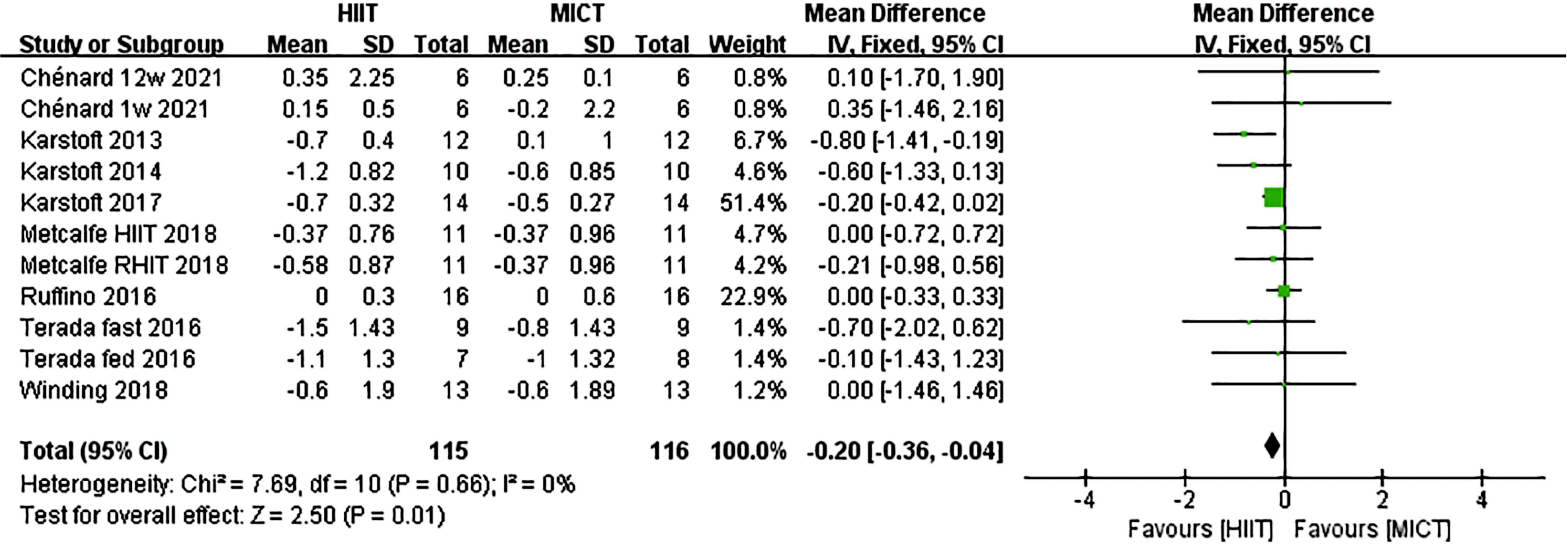
Effect of HIIT versus MICT on mean 24-h glucose. HIIT, high-intensity interval training; MICT, moderate-intensity continuous training; REHIT, reduced-exertion high-intensity interval training; 1HIIT, 1-min interval HIIT; 4HIIT, 4-min interval HIIT; 1w, after 1 week; 12w, after 12 weeks; m, morning; n, afternoon/night as reported in the original study.

For the HIIT *vs*. MICT comparison, 11 comparison units were included in the meta-analysis. Heterogeneity was low (Chi^2^ = 7.69, p = 0.66; I^2^ = 0%), and therefore a fixed-effect model was used. The pooled analysis showed that, compared with MICT, HIIT further reduced 24-hour mean glucose (MD = -0.20 mmol/L, 95% CI -0.36 to -0.04, p = 0.01; **Figure 3**). Leave-one-out sensitivity analyzes suggested that the statistical significance of the overall effect may change after exclusion of Karstoft 2013 or Karstoft 2017, indicating that the result remained somewhat sensitive to individual studies. Funnel plot inspection did not suggest obvious publication bias (**Supplementary Figure 2B**). The certainty of evidence for this comparison was rated as low by GRADE (**Supplementary Table 4**).

Individual study findings suggested that implementation conditions may influence the magnitude of change in 24-hour mean glucose. For example, in the study by Terada et al., the changes in 24-hour mean glucose following HIIT performed under fasted and postprandial conditions were −1.5 ± 0.8 mmol/L and −1.1 ± 0.6 mmol/L, respectively (22). In the study by Savikj et al., HIIT performed in the afternoon showed a more favorable glucose change trend compared to HIIT performed in the morning (23). In the study by Metcalfe et al., there were also differences in the changes in 24-hour mean glucose following REHIT and traditional HIIT (24). Additionally, the supplementary material of Marcotte-Chénard et al. (25) showed that both HIIT1 and HIIT4 were associated with lower 24-hour mean glucose than the non-exercise control condition. Because valid paired effect estimates or other variance information suitable for crossover-based quantitative pooling were unavailable for this outcome, these findings were summarized narratively rather than included in the pooled analysis (25).

After analysis by predefined subgroup variables, the complete subgroup results for HIIT *vs*. CON and HIIT *vs*. MICT are provided in **Supplementary Table 5**. Overall, the subgroup differences in the HIIT *vs*. CON comparison were statistically significant, suggesting that BMI, intervention duration, or CGM monitoring window may be related to effect size, though the related results should be interpreted with caution. No statistically significant differences were observed in the HIIT *vs*. MICT comparison.

#### 3.4.2 Postprandial glucose

**Figure 4:**
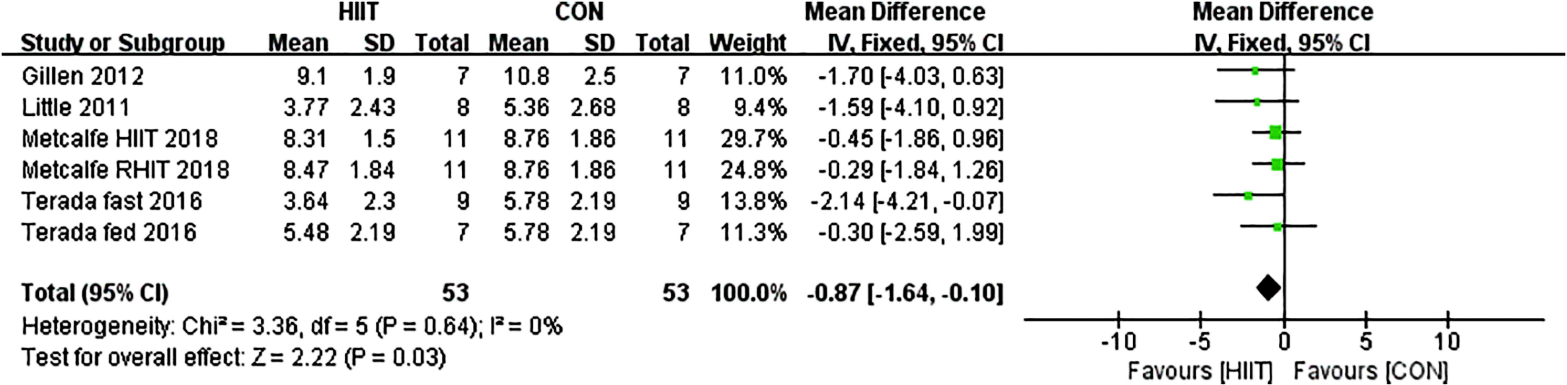
Effect of HIIT versus CON on postprandial glucose. HIIT, high-intensity interval training; REHIT, reduced-exertion high-intensity interval training.

For the HIIT *vs*. CON comparison, 4 studies contributing 6 comparison units were included in the meta-analysis. Between-study heterogeneity was low (Chi^2^ = 3.36, p = 0.64; I^2^ = 0%), and therefore a fixed-effect model was applied. The pooled analysis showed that, compared with non-exercise control, HIIT significantly reduced postprandial glucose (MD = -0.87 mmol/L, 95% CI -1.64 to -0.10, p = 0.03; **Figure 4**). Leave-one-out sensitivity analyzes indicated that exclusion of Little 2011, Gillen 2012, or Terada (fast) 2016 did not materially change heterogeneity, but the statistical significance of the pooled effect could change, suggesting that the result was relatively sensitive to individual studies or comparison units. Because fewer than 10 comparison units were available, publication bias was not further assessed according to the prespecified criteria. According to the GRADE assessment, the certainty of evidence for this outcome was low (**Supplementary Table 4**).

For the HIIT *vs*. MICT comparison, 2 studies contributing 4 comparison units reported postprandial glucose results that were potentially relevant for comparison. Of these, three comparison units favored MICT, whereas one comparison unit favored HIIT. Because the amount of combinable data was limited and the intervention protocols differed substantially across studies, no further meta-analysis was performed, and this comparison was summarized narratively only.

Individual study findings suggested that implementation conditions may influence the magnitude of change in postprandial glucose. For example, in the study by Terada et al., the changes in postprandial glucose following HIIT performed under fasted and postprandial conditions were -1.43 ± 1.54 mmol/L and 0.20 ± 1.32 mmol/L, respectively (22). In the study by Metcalfe et al., the changes in postprandial glucose following REHIT and traditional HIIT were -0.29 ± 0.82 mmol/L and -0.37 ± 0.37 mmol/L, respectively (24). These findings suggest that feeding status and specific HIIT structure may modify the effects of HIIT on postprandial glucose.

#### 3.4.3 MAGE

The GV metrics reported in the included studies included MAGE and coefficient of variation (CV%), among others. Because MAGE was reported more consistently and met the prespecified criteria for pooling, a meta-analysis was performed for MAGE (mmol/L).

**Figure 5:**
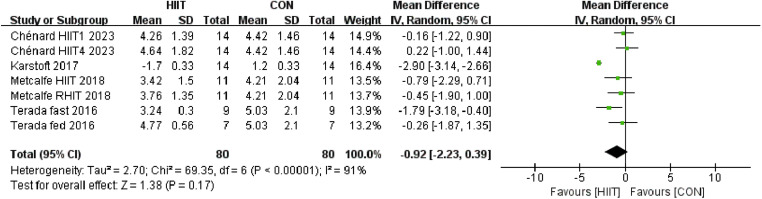
Effect of HIIT versus CON on MAGE.

For the HIIT *vs*. CON comparison, 4 studies contributing 7 comparison units were included in the meta-analysis. Due to substantial heterogeneity between studies (Chi^2^ = 69.35, p< 0.00001; I^2^ = 91%), a random-effects model was applied. The pooled analysis showed that, compared with non-exercise control, no statistically significant difference was observed for MAGE (MD = −0.92 mmol/L, 95% CI −2.23 to 0.39, p = 0.17; **Figure 5**). Leave-one-out sensitivity analyzes showed that, after exclusion of Karstoft 2017, heterogeneity decreased to I^2^ = 5%, but the overall conclusion did not change significantly. Further exclusion of Metcalfe REHIT 2018 or Terada (fed) 2016 could also alter the overall conclusion, although the impact on heterogeneity was limited. Because fewer than 10 comparison units were available, publication bias was not further assessed according to the prespecified criteria. Taking into account the risk of bias, substantial heterogeneity, and limited sample size, the GRADE assessment rated the certainty of evidence for this outcome as very low (**Supplementary Table 4**).

**Figure 6:**
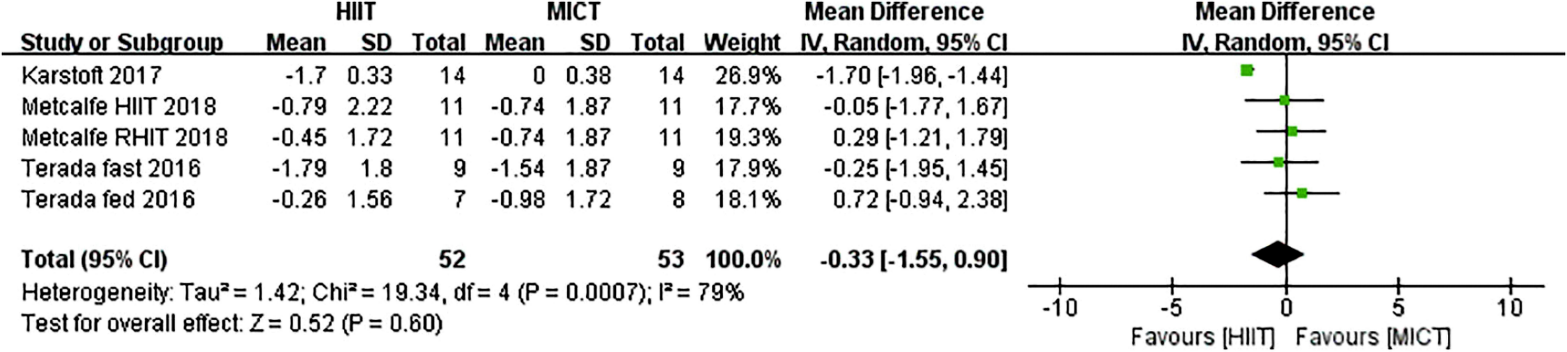
Effect of HIIT versus MICT on MAGE. HIIT, high-intensity interval training; MICT, moderate-intensity continuous training; REHIT, reduced-exertion high-intensity interval training; MAGE, mean amplitude of glycemic excursions. 1HIIT, 1-min interval HIIT; 4HIIT, 4-min interval HIIT.

For the HIIT *vs*. MICT comparison, 3 studies contributing 5 comparison units were included in the meta-analysis. Between-study heterogeneity was substantial (Chi^2^ = 19.34, p = 0.0007; I^2^ = 79%), and therefore a random-effects model was used. The pooled analysis showed that, compared with MICT, HIIT had no statistically significant effect on MAGE (MD = -0.33 mmol/L, 95% CI -1.55 to 0.90, p = 0.60; **Figure 6**). Leave-one-out sensitivity analyzes indicated that exclusion of Karstoft 2017 markedly reduced heterogeneity, but the overall conclusion did not materially change, suggesting that the direction of the effect was relatively stable, although still influenced by between-study differences. Because fewer than 10 comparison units were available, publication bias was not further assessed. The certainty of evidence for this comparison was rated as very low by GRADE (**Supplementary Table 4**).

In addition, some individual studies suggested that implementation-related factors may influence the magnitude of change in MAGE. For example, in the study by Terada et al., the changes in MAGE following HIIT performed under fasted and postprandial conditions were −1.79 ± 1.80 mmol/L and −0.26 ± 1.56 mmol/L, respectively (22). In the study by Metcalfe et al., the changes in MAGE following REHIT and traditional HIIT were −0.45 ± 1.72 mmol/L and −0.79 ± 0.22 mmol/L, respectively (24). In addition, supplementary data from Marcotte-Chénard et al. (25) showed that MAGE values in the overall sample were 4.38 ± 1.66, 4.24 ± 2.71, and 4.56 ± 2.31 mmol/L under the HIIT1, HIIT4, and control conditions, respectively, with no statistically significant overall difference. In the subgroup with higher baseline glycemia, the reduction in MAGE appeared more pronounced under HIIT1, whereas a similar pattern was not observed under HIIT4. For outcomes or comparisons in Marcotte-Chénard et al. (2023) that did not provide effect estimates or variance information suitable for crossover-based quantitative pooling, the findings were summarized narratively (25). Due to the high heterogeneity between studies, the inclusion of these studies had limited contribution to the quantitative results.

#### 3.4.4 Time above range and time below range

##### 3.4.4.1 TAR

**Figure 7:**
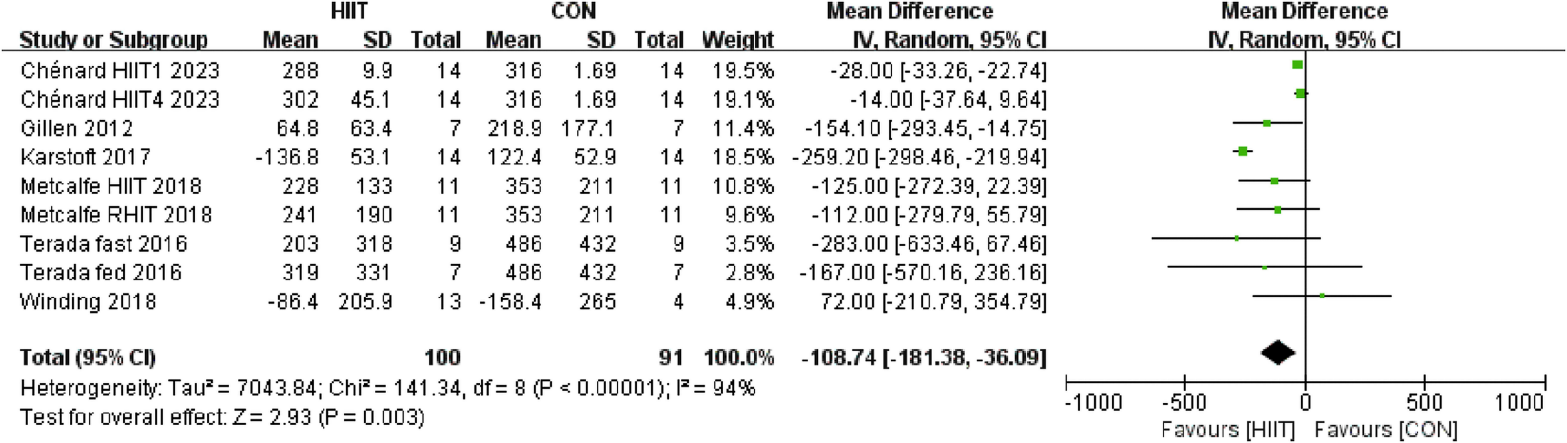
Effect of HIIT versus CON on TAR.

For the HIIT *vs*. CON comparison, 6 studies contributing 9 comparison units were included in the meta-analysis. The heterogeneity between studies was high (Chi^2^ = 141.34, p< 0.00001; I^2^ = 94%), so a random-effects model was applied. The pooled analysis showed that, compared with non-exercise control, HIIT significantly reduced TAR (MD = −108.74 min, 95% CI −181.38 to −36.09, p = 0.003; **Figure 7**). Leave-one-out sensitivity analyzes showed that, after excluding the Chénard HIIT1–2023 study, the overall direction of effect changed substantially, indicating that this study had a large impact on the results. Since fewer than 10 comparison units were available, publication bias was not further assessed according to the prespecified criteria. Taking into account the risk of bias, limited sample size, and differences in study design, the GRADE assessment rated the certainty of evidence for this outcome as low (**Supplementary Table 4**).

**Figure 8:**
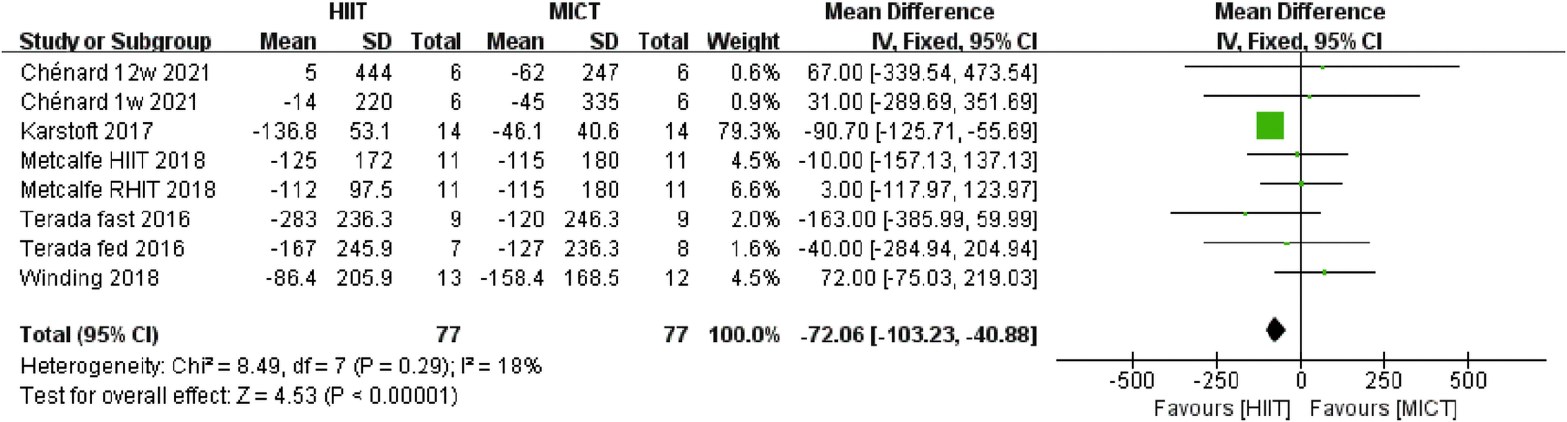
Effect of HIIT versus MICT on TAR. HIIT, high-intensity interval training; MICT, moderate-intensity continuous training; REHIT, reduced-exertion high-intensity interval training; TAR, time above range; TBR, time below range; HIIT1, 1-min interval HIIT; HIIT4, 4-min interval HIIT; 1w, after 1 week; 12w, after 12 weeks.

For the HIIT *vs*. MICT comparison, 5 studies contributing 8 comparison units were included in the meta-analysis. Between-study heterogeneity was low (Chi^2^ = 8.49, p = 0.29; I^2^ = 18%), and therefore a fixed-effect model was used. The pooled analysis showed that, compared with MICT, HIIT further reduced TAR (MD = -72.06 min, 95% CI -103.23 to -40.88, p< 0.0001; **Figure 8**). Leave-one-out sensitivity analyzes indicated that exclusion of Karstoft 2017 could change the statistical significance of the overall effect, suggesting that the result remained somewhat sensitive to individual studies. Because fewer than 10 comparison units were available, publication bias was not further assessed. The certainty of evidence for this comparison was rated as low by GRADE (**Supplementary Table 4**).

In addition, some individual studies suggested that implementation-related factors may influence the magnitude of change in TAR. For example, in the study by Terada et al., the changes in TAR following HIIT performed under fasted and postprandial conditions were −283 ± 236 min and −167 ± 246 min, respectively (22). In the study by Metcalfe et al., the changes in TAR following REHIT and traditional HIIT were −112 ± 98 min and −125 ± 172 min, respectively (24). Additionally, supplementary data from Marcotte-Chénard et al. (2023) showed that TAR was lower under both HIIT1 and HIIT4 conditions compared to the control condition in the overall sample. This contrast appeared more pronounced in the subgroup with higher baseline glycemia, with a relatively greater reduction under HIIT1. For outcomes or comparisons in Marcotte-Chénard et al. (2023) that did not provide effect estimates or variance information suitable for crossover-based quantitative pooling, the findings were summarized narratively (25). Due to high heterogeneity between studies, the inclusion of these studies contributed minimally to the quantitative results.

##### 3.4.4.2 TBR

TBR was reported less frequently, and substantial differences existed across studies in terms of hypoglycemia thresholds, reporting formats, and event definitions. Therefore, the available data did not meet the prespecified criteria for meta-analysis. A small number of studies reported changes in TBR before and after intervention, but the findings were inconsistent. In comparisons of HIIT versus CON, some studies observed increases in TBR under either the HIIT or control condition (26, 27). In comparisons of HIIT versus MICT, some studies also suggested that the magnitude of change in TBR differed between groups (26, 27).

In addition, supplementary data from Marcotte-Chénard et al. (2023) showed that TBR was lower under both HIIT1 and HIIT4 conditions compared to the control condition in the overall sample, and this contrast appeared more pronounced in the subgroup with higher baseline glycemia, with a relatively greater reduction under HIIT1. However, a similar change was not observed under HIIT4 (25). These findings further support differences in the effects of various HIIT conditions on TBR. One study also reported that no hypoglycemic events occurred under the MICT condition, while a small number of participants in the HIIT condition experienced brief hypoglycemia the day after training (28). Given the limited amount of evidence and the inconsistency in thresholds and analytical approaches across studies, these findings should be interpreted with caution.

## 4 Discussion

The present systematic review and meta-analysis suggests that HIIT may have favorable effects on selected CGM-derived outcomes in adults with T2D, although the strength of evidence differed across outcomes. Overall, compared with non-exercise control, HIIT showed a more consistent favorable direction of effect for PPG and TAR, whereas the findings for 24-hour mean glucose should still be interpreted cautiously in light of heterogeneity and limited certainty of evidence. Compared with MICT, HIIT showed advantages in reducing 24-hour mean glucose and TAR. By contrast, the evidence for glycemic variability, particularly as represented by MAGE, remained inconsistent, and evidence for TBR was limited and insufficiently standardized. Taken together, current evidence more strongly supports a potential role for HIIT in improving mean glycemic exposure and hyperglycemic burden than in improving all CGM-derived outcomes more broadly.

The interpretation of these findings should also consider that the included evidence base comprised both acute single-session studies and longer-term training interventions. Acute studies are more likely to reflect short-term effects on glycemic exposure over the subsequent hours or day, whereas longer-term interventions may additionally reflect training adaptation, behavioral change, and possible body-composition effects. These different timescales should not be interpreted as equivalent.

### 4.1 Effect of HIIT on 24-hour mean glucose

The finding for 24-hour mean glucose should be interpreted as a condition-dependent signal rather than as a uniform effect of HIIT across all implementation settings. The direction of effect generally favored HIIT, but the magnitude of change varied across studies and was influenced by protocol structure, feeding status, exercise timing, baseline glycemic status, and CGM monitoring window. This variability is clinically important because acute single-session studies primarily capture short-term post-exercise glycemic exposure, whereas longer training interventions may additionally reflect adaptations in fitness, habitual activity, and body composition.

From a physiological perspective, the short-term reduction in mean glucose after HIIT is biologically plausible. Muscle contraction can stimulate glucose uptake through insulin-independent pathways involving AMPK- and calcium-related signaling and GLUT4 translocation to the sarcolemma (29, 30). During recovery, exercise-induced glycogen depletion and the subsequent need for glycogen resynthesis may further increase skeletal-muscle glucose disposal and insulin sensitivity (29, 30). HIIT may differ from MICT because repeated high-intensity work intervals can generate greater transient metabolic stress, recruit a larger proportion of type II muscle fibers, and produce substantial local glycogen turnover within a shorter time. By contrast, MICT may rely more on longer cumulative exercise duration, sustained oxidative metabolism, and total energy expenditure. These mechanisms are not mutually exclusive, and the relative contribution of intensity and volume probably depends on the protocol and participant characteristics.

However, the mechanistic interpretation of the present findings should remain cautious. Most included CGM studies did not directly measure insulin sensitivity, muscle glycogen, GLUT4 signaling, substrate oxidation, or catecholamine responses. Therefore, the proposed mechanisms provide biological plausibility but do not establish direct mechanistic mediation for the CGM-derived outcomes observed in this review.

Further research is therefore needed to clarify which HIIT protocols, under which implementation conditions, are most likely to improve 24-hour mean glucose in adults with T2D. Future studies should consider intervention intensity, duration, interval structure, exercise timing, feeding status, baseline glycemic control, and CGM monitoring windows when evaluating the effects of HIIT on daily glycemic exposure.

### 4.2 Effect of HIIT on PPG

PPG is an important indicator of postprandial glucose handling and is clinically relevant because it contributes meaningfully to overall glycemic exposure and cardiovascular risk (31, 32). In the present review, HIIT was associated with lower PPG compared with non-exercise control, which is broadly consistent with previous evidence suggesting that high-intensity interval exercise may reduce postprandial glycemic exposure (33–35). Compared with 24-hour mean glucose, PPG may be more sensitive to changes in energy metabolic state before and after exercise, muscle glycogen utilization, and subsequent glucose disposal, and may therefore be more responsive to single-session or short-term HIIT in theory. Mechanistically, the potential benefit of HIIT for PPG may be related to enhanced skeletal muscle glucose uptake, improved postprandial glucose clearance, and increased demand for glycogen resynthesis. However, direct evidence linking these mechanisms to CGM-derived postprandial outcomes in adults with T2D remains limited, and the included studies did not consistently report concurrent metabolic measures. Accordingly, mechanistic interpretation should remain cautious.

By contrast, comparative evidence for HIIT versus MICT with respect to PPG remains limited and inconsistent. This inconsistency may relate to differences in total exercise dose, energy expenditure, feeding status, the timing of exercise relative to meals, and variation in the definition and observation window of PPG across studies (22, 24, 36). Therefore, current evidence supports a potential benefit of HIIT for PPG compared with non-exercise control, but does not yet demonstrate a stable advantage over MICT.

A small number of exploratory studies further suggest that PPG may be particularly sensitive to implementation conditions. For example, fasted versus postprandial exercise, as well as differences in HIIT/REHIT structure, may lead to different magnitudes of change in PPG (22, 24, 37). These findings suggest that PPG may be especially responsive to exercise prescription characteristics and support a shift in future research from asking whether HIIT is beneficial to clarifying which HIIT configuration, under which conditions, is most effective for reducing postprandial glucose. Larger randomized controlled trials with standardized postprandial assessment windows, controlled meal conditions, and clearly reported intervention timing will be important for improving comparability across studies and for identifying the most effective prescription characteristics.

### 4.3 Effect of HIIT on GV

GV is considered an important dimension in evaluating the value of exercise interventions (38), but its metric system is relatively complex and has not yet been fully standardized (5). Because the GV metric that most consistently met the criteria for pooling in the present review was MAGE, the current conclusions are more accurately interpreted as relating to the effect of HIIT on MAGE rather than on GV as a whole.

Based on the pooled results, the primary analyzes for MAGE did not provide stable and consistent evidence of benefit for HIIT compared with either non-exercise control or MICT, and heterogeneity was substantial in both comparisons. Although the comparison versus non-exercise control showed a trend toward significance, sensitivity analyzes indicated that the conclusion was sensitive to individual studies (6). Thus, current evidence does not support a stable beneficial effect of HIIT on glycemic variability as represented by MAGE.

Several factors may contribute to this inconsistency. First, MAGE preferentially captures larger-amplitude glucose excursions and may be particularly sensitive to hyperglycemic fluctuations, but it may not fully represent all forms of within-day glycemic instability. Second, compared with 24-hour mean glucose, MAGE may be more sensitive to differences in monitoring duration, dietary control, habitual activity, and baseline metabolic status, thereby making between-study variation more likely. Third, differences in HIIT prescription characteristics, including work-interval duration, recovery structure, total exercise dose, and timing of implementation, may influence both immediate post-exercise fluctuations and subsequent recovery-phase glycemic patterns (22, 24, 25).

Accordingly, a more appropriate interpretation at this stage is that current evidence is insufficient to demonstrate that HIIT can stably improve glycemic variability as represented by MAGE, but this should be understood as reflecting limited evidence and substantial heterogeneity rather than firm evidence of no effect. Future studies should prioritize more standardized reporting of GV outcomes, including MAGE, CV, and key fluctuation-related metrics derived from TIR, TAR, and TBR, together with greater transparency in CGM data processing and statistical analysis, in order to better characterize the true effects of HIIT on glycemic fluctuation patterns.

### 4.4 Effect of HIIT on TAR and TBR

TAR provides a relatively direct reflection of the burden of hyperglycemic exposure and is therefore a clinically meaningful CGM-derived outcome (32, 39). Based on the pooled results of the present review, HIIT reduced TAR compared with non-exercise control and also showed a favorable effect compared with MICT. These findings suggest that the potential value of HIIT may extend beyond modest reductions in mean glucose to include reductions in the duration of clinically relevant hyperglycemia (40).

Notably, TAR appeared more consistent than several other CGM-derived outcomes, particularly in the comparison with MICT, where between-study heterogeneity was low. This pattern suggests that TAR may more readily and stably reflect the intervention value of HIIT than some other CGM metrics. Even so, differences across studies in medication use, meal control, physical activity restrictions, and exercise prescription should temper interpretation (22, 24, 27). Accordingly, the current evidence more strongly supports the conclusion that HIIT may help reduce TAR, rather than the stronger claim that it is uniformly superior under all implementation conditions.

By contrast, evidence for TBR was clearly insufficient, and findings across studies were inconsistent (41). Some studies reported increased TBR following HIIT (26, 27), whereas others observed brief hypoglycemic events in a small number of participants after HIIT (28). However, the current evidence base remains limited by the small number of studies, inconsistent hypoglycemia thresholds, variation in reporting formats and analytical approaches, and incomplete event reporting (42, 43). Therefore, no stable conclusion can yet be drawn regarding the effect of HIIT on hypoglycemia risk.

From a clinical perspective, this issue remains important, especially in individuals using glucose-lowering medications or those with greater baseline glycemic instability. Future studies should report TBR and hypoglycemic events more systematically in order to improve assessment of the balance between potential benefits and risks.

### 4.5 Interpretation of CGM-derived outcomes and methodological considerations

Interpretation of the present findings should take into account the structure of CGM metrics and several related methodological limitations. First, the CGM-derived outcomes reported across studies were not fully standardized, particularly for GV-related metrics. Accordingly, the current evidence is better interpreted as supporting the possibility of improvements in selected CGM-derived outcomes rather than comprehensive improvements across all CGM outcomes. In particular, because the pooled GV outcome in this review was primarily MAGE, the relevant conclusions should specifically concern the effect of HIIT on MAGE, rather than extrapolating the results to all aspects of glycemic variability.

Second, although the search covered the period from database inception to January 26, 2026, the most recent eligible included study was published in 2023. No controlled HIIT intervention studies published in 2024, 2025, or January 2026 met all eligibility criteria after screening, including adult T2D population, structured HIIT intervention, controlled design, and CGM-derived outcome reporting with usable data. The included studies therefore spanned 2011-2023, a period during which CGM technology underwent substantial evolution. Differences in sensor performance, calibration requirements, data availability, user experience, and data processing software may have influenced comparability across studies and may represent an additional source of heterogeneity.

Third, although CGM is commonly described as “continuous,” it still records interstitial glucose at fixed time intervals, and interstitial glucose also lags physiologically behind blood glucose. This limitation is especially relevant for HIIT, which often involves short work and recovery bouts that occur on a timescale shorter than can be precisely captured by CGM. Accordingly, CGM is more suitable for evaluating overall glycemic exposure over the subsequent minutes, hours, or day after HIIT, rather than for precisely characterizing glucose responses within each brief interval. Therefore, interpretation should clearly distinguish between short-term dynamic responses during exercise and overall glycemic exposure after exercise.

Another methodological issue concerns exercise-intensity monitoring. Several included studies prescribed or monitored exercise intensity using HR-based targets. This approach is practical but may be affected by beta-blocker use, which is common in adults with T2D and cardiovascular comorbidities and can attenuate the HR response to exercise. In the included studies, medication use was not consistently reported in sufficient detail to determine whether HR targets were adjusted for beta-blocker therapy or whether analyzes accounted for beta-blocker use. This limitation may have contributed to variability in the achieved exercise intensity and should be considered when interpreting HR-based HIIT protocols. Future studies should report beta-blocker use, specify whether exercise testing and HR targets were determined under usual medication conditions, and, where feasible, combine HR monitoring with RPE, workload or power output, speed/grade, or oxygen uptake. Comparator availability should also be considered. Resistance exercise and combined aerobic-resistance exercise were eligible in principle if they were directly compared with HIIT and reported usable CGM-derived outcomes in adults with T2D. However, the eligible studies identified in this review provided comparative CGM data mainly for HIIT versus non-exercise control and HIIT versus MICT. No eligible study directly comparing HIIT with resistance exercise provided sufficient CGM-derived outcome data for quantitative synthesis. Therefore, evidence directly comparing the glycemic effects of HIIT and resistance exercise using CGM-derived outcomes remains limited.

Finally, the structure of the available evidence imposes additional limitations. Some acute crossover trials did not provide valid paired effect estimates suitable for quantitative pooling, restricting their inclusion in the meta-analyzes. In addition, several outcomes were informed by only a small number of studies or comparison units, which limited the interpretability of funnel plots, subgroup analyzes, and mechanism-based inference. Future research should improve the reporting of paired effect estimates, change scores, and variance measures in crossover trials to enhance the reliability of future evidence synthesis. Although international consensus statements have clearly defined core CGM metrics such as TIR, TAR, and TBR, heterogeneity persists across studies in the selection of CGM-derived outcomes, calculation methods, analysis time windows, device-related monitoring protocols, and reporting frameworks, particularly for glycemic variability metrics.

### 4.6 Strengths and limitations

The present review has several strengths. First, it specifically focused on the effects of HIIT on CGM-derived outcomes in adults with T2D. Compared with studies focusing only on conventional markers such as fasting blood glucose or HbA1c, the present review evaluates the potential effects of exercise interventions across multiple dimensions, including overall daily glycemic exposure, postprandial glucose responses, and hyperglycemic and hypoglycemic exposure. Second, whenever data were available, this review prioritized the pooling of change-from-baseline values and separately compared HIIT with non-exercise controls and with other exercise interventions, which helps improve the specificity and interpretability of the findings. Third, the review applied the GRADE approach to assess the certainty of evidence for the major outcomes, allowing interpretation to extend beyond statistical significance alone and to incorporate the strength of evidence and its potential clinical relevance. In addition, this review systematically summarized CGM-related methodological characteristics across studies, including device brand/model, monitoring window, wear duration, and the extent to which medication use, dietary intake, and physical activity were controlled or recorded during the monitoring period. This helps clarify important design differences across studies.

Several limitations should also be acknowledged. First, the available evidence was limited by small sample sizes, and most included studies were small trials. Inadequate reporting of random sequence generation and allocation concealment was common. Because of the nature of exercise interventions, blinding of participants and personnel is inherently difficult, and these issues were insufficiently reported in many studies, which may reduce confidence in the effect estimates. Second, there was heterogeneity in CGM monitoring duration, the interval between the last exercise session and CGM assessment, and the extent to which medication use, dietary intake, habitual physical activity, and exercise timing were controlled or recorded. Third, medication-related information, especially beta-blocker use and its implications for HR-based intensity prescription, was inconsistently reported. Fourth, no eligible HIIT versus resistance-exercise comparison with usable CGM-derived data was identified. These limitations should be considered when interpreting the certainty and applicability of the findings.

Overall, the present review provides evidence-informed insight into the potential effects of HIIT on selected CGM-derived outcomes in adults with T2D, but at this stage the evidence should be regarded as informative rather than definitive.

## 5 Conclusions and practical implications

Overall, current evidence suggests that, compared with non-exercise control, HIIT may improve selected CGM-derived outcomes in adults with T2D, particularly PPG and TAR. Compared with MICT, HIIT may offer some additional benefit in reducing 24-hour mean glucose and TAR. By contrast, the evidence regarding glycemic variability and time below range remains inconsistent or limited. Accordingly, current findings more strongly support a potential role for HIIT in improving selected hyperglycemia-related outcomes than in producing stable improvement across all CGM-derived outcomes.

From a clinical perspective, CGM provides a practical framework for evaluating the effects of exercise interventions across multiple dimensions, including overall daily glycemic exposure, postprandial responses, and potential hypoglycemia-related risk. It may therefore support the development of more individualized exercise prescriptions. With appropriate patient education, risk screening, and safety management, HIIT may serve as an alternative or complementary exercise option for selected adults with T2D, particularly for those seeking meaningful reductions in hyperglycemic exposure within a relatively short time commitment. However, because the available evidence remains limited by small sample sizes, heterogeneity in study design and intervention protocols, and the generally low certainty of evidence for several key outcomes, these conclusions should be interpreted cautiously and not overgeneralized.

Future studies should move beyond small and heterogeneous trials by using adequately powered randomized designs and standardized CGM reporting procedures. Specifically, investigators should prespecify CGM-derived outcomes, standardize CGM wear duration and analysis windows, apply consensus threshold definitions where applicable, and clearly report meal timing and composition, medication use, beta-blocker status, baseline physical activity, and exercise timing relative to CGM assessment. HIIT protocols should also be described in sufficient detail, including intensity anchors, work-interval duration, recovery mode, work-to-recovery ratio, session duration, weekly frequency, progression rules, supervision, and total intervention duration. Longer interventions are needed to determine whether short-term CGM improvements translate into durable glycemic and cardiometabolic benefits. Future trials should also include clinically relevant head-to-head comparisons between HIIT, MICT, resistance training, and combined aerobic-resistance exercise. For crossover trials, paired effect estimates, change scores, and corresponding variance measures should be reported to facilitate appropriate quantitative synthesis.
