# Supplementary material for: High-intensity interval training and continuous glucose monitoring-derived glycemic outcomes in adults with type 2 diabetes: a systematic review and meta-analysis
Source: Front Endocrinol (Lausanne). 2026 Jun 17;17:1834479. doi: 10.3389/fendo.2026.1834479 (PMC13318697; doi:10.3389/fendo.2026.1834479)
Supplement: Supplementary file 1 [file DataSheet1.docx]

| Supplementary Figure1. |
| --- |
| 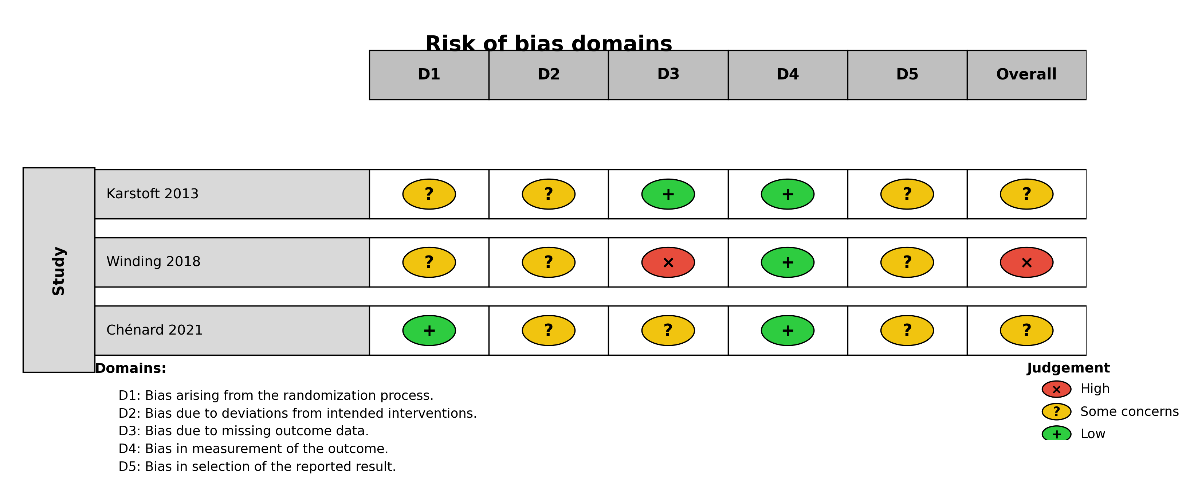 |
| Figure Sa-1. Risk of bias assessment of randomized parallel trials using RoB 2. |
| 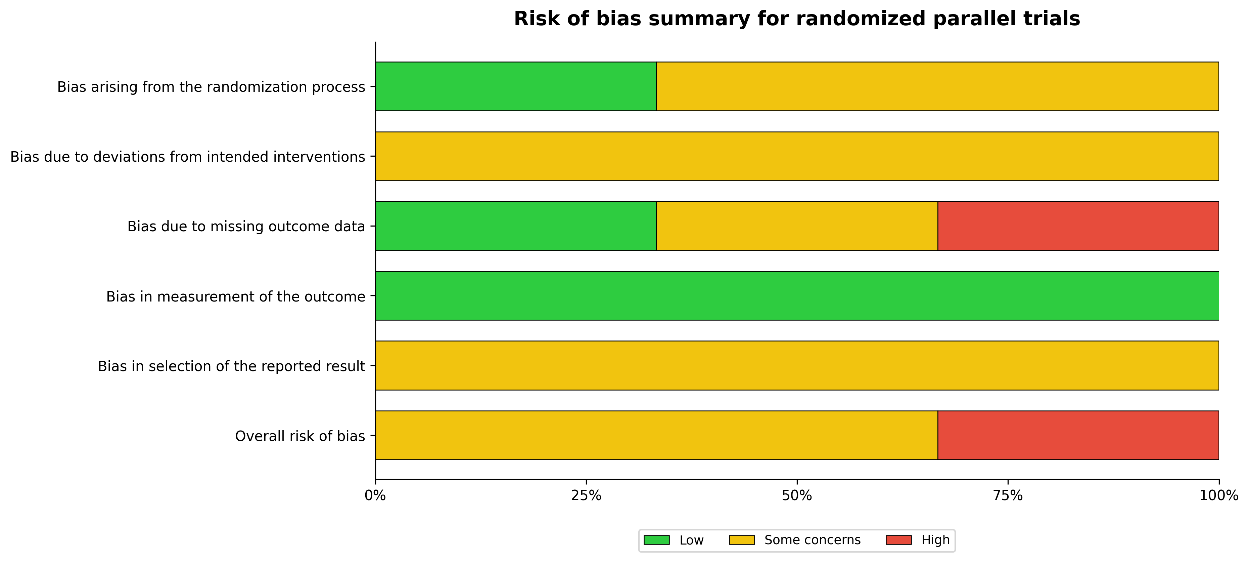 |
| Figure Sa-2. Distribution of risk-of-bias judgments across randomized parallel trials using RoB 2. |
|  |
| 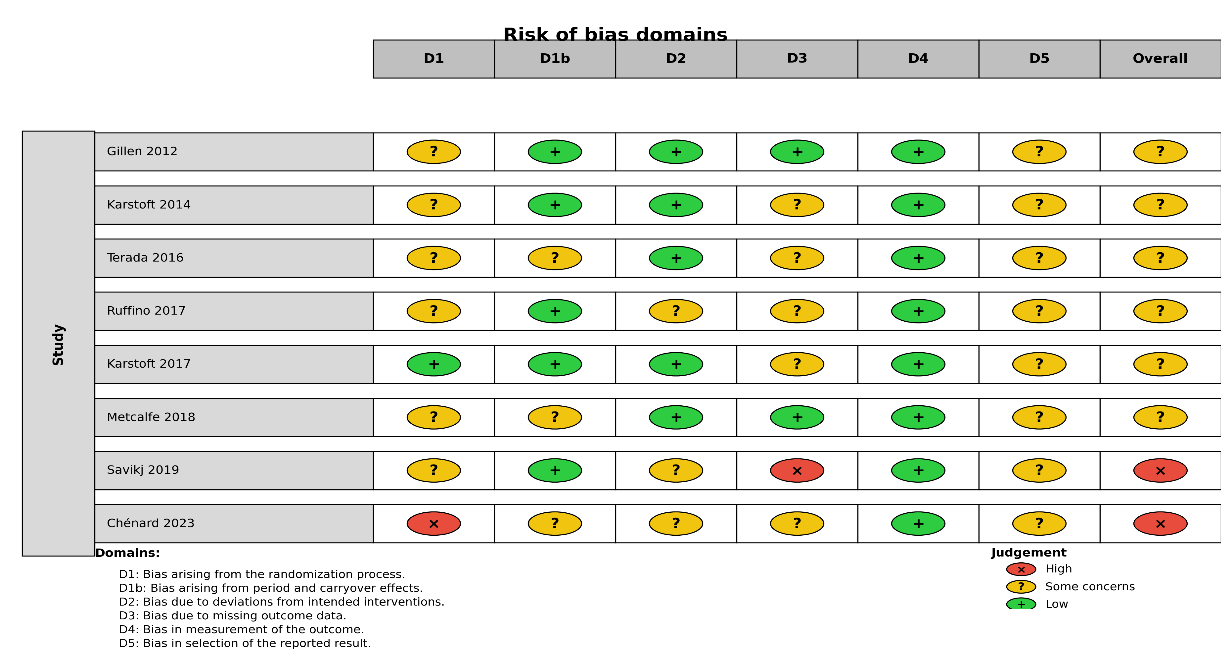 |
| Figure Sb-1. Risk of bias assessment of crossover trials using RoB 2 for crossover trials. |
| 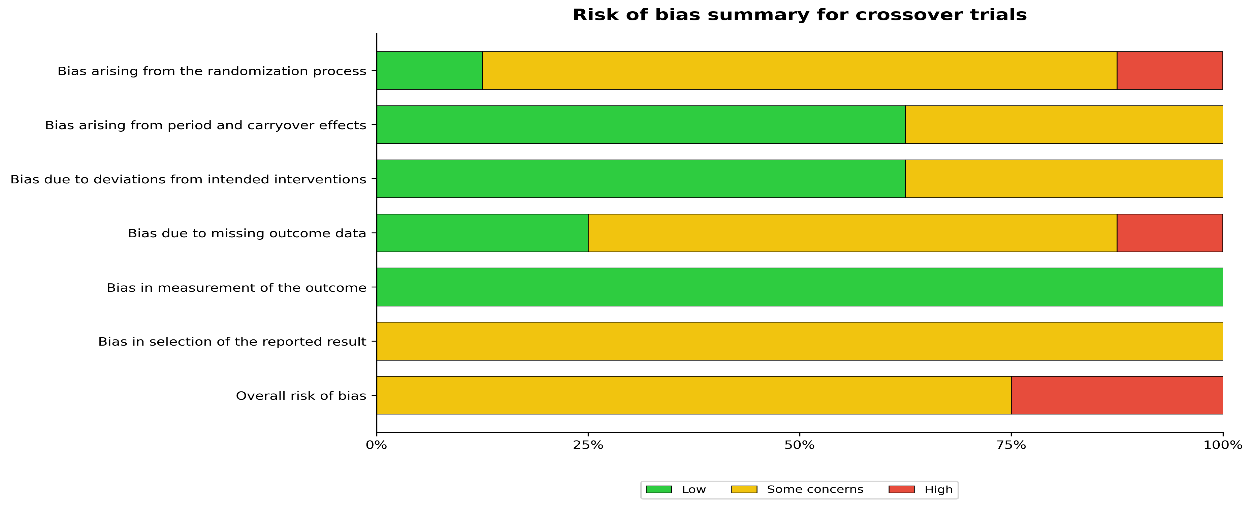 |
| Figure Sb-2. Distribution of risk-of-bias judgments across crossover trials using RoB 2 for crossover trials. |
|  |
| 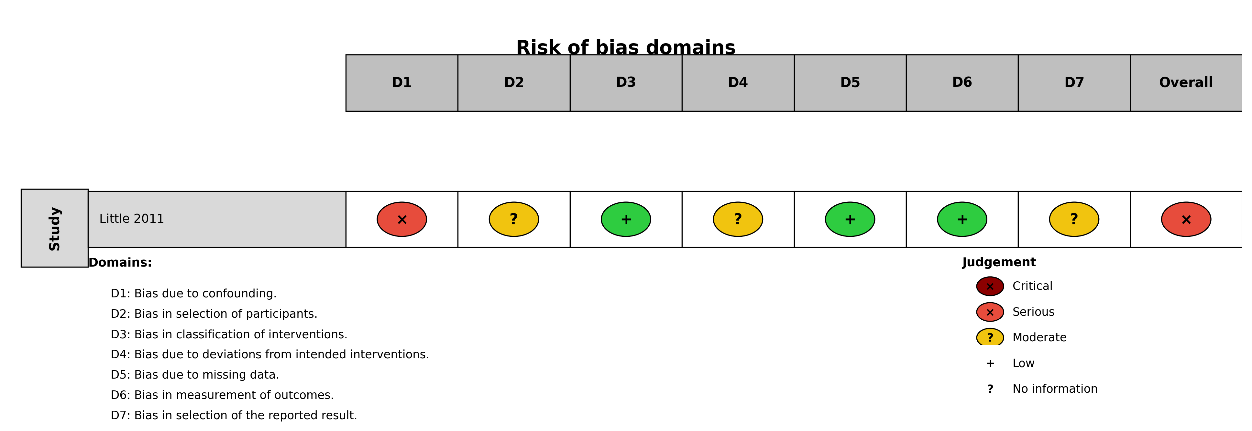 |
| Figure Sc-1. Risk of bias assessment of the non-randomized intervention study using ROBINS-I. |
| 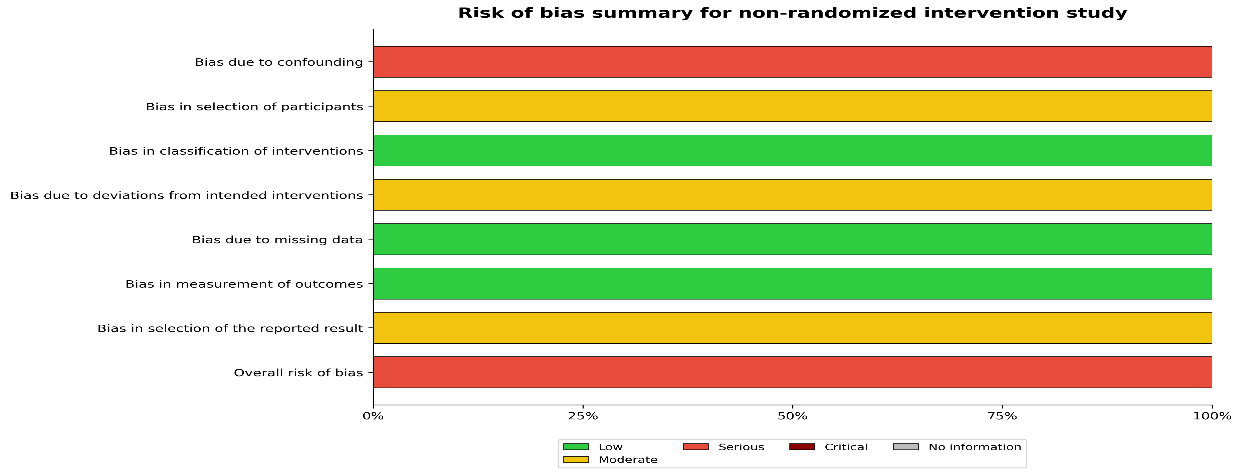 |
| Figure Sc-2. Distribution of risk-of-bias judgments for the non-randomized intervention study using ROBINS-I. |
