# Supplementary material for: High-intensity interval training and continuous glucose monitoring-derived glycemic outcomes in adults with type 2 diabetes: a systematic review and meta-analysis
Source: Front Endocrinol (Lausanne). 2026 Jun 17;17:1834479. doi: 10.3389/fendo.2026.1834479 (PMC13318697; doi:10.3389/fendo.2026.1834479)
Supplement: Supplementary file 2 [file DataSheet2.docx]

Supplementary Figure S2.

| 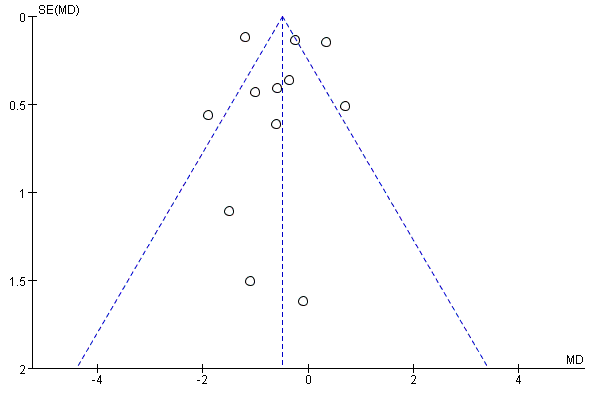 |
| --- |
| **Figure S2a. Comparison of 24h mean glucose between HIIT and CON** |
|  |
| 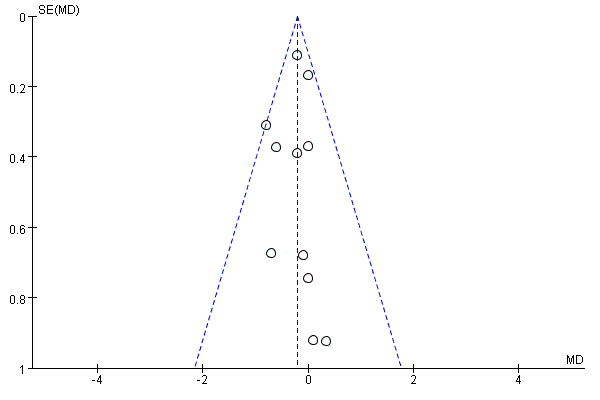 |
| **Figure S2b. Comparison of 24h mean glucose between HIIT and MICT** |
