# Supplementary material for: High-intensity interval training and continuous glucose monitoring-derived glycemic outcomes in adults with type 2 diabetes: a systematic review and meta-analysis
Source: Front Endocrinol (Lausanne). 2026 Jun 17;17:1834479. doi: 10.3389/fendo.2026.1834479 (PMC13318697; doi:10.3389/fendo.2026.1834479)
Supplement: Supplementary file 3 [file DataSheet3.docx]

**Supplementary Table S1.**

**Detailed search strategy for all databases**

**Search overview**

A comprehensive electronic search was conducted in PubMed (MEDLINE), Embase, Web of Science Core Collection, Cochrane Library, and SPORTDiscus (via EBSCOhost) from database inception to 26 January 2026. Searches were limited to English-language records. The strategy combined controlled vocabulary (e.g., MeSH and Emtree terms) and free-text keywords related to type 2 diabetes, continuous glucose monitoring, and high-intensity interval training. To improve retrieval of eligible experimental studies, an additional sensitive free-text filter related to interventional study design was applied. Reference lists of all included studies were also manually screened to identify any additional eligible trials not captured by the electronic searches.

During manuscript revision, additional sensitivity searches were conducted across all originally searched databases to assess the completeness of the original search strategy in response to peer-review comments.

Core concepts and search terms

1. Population: adults with type 2 diabetes

Controlled vocabulary (examples):

PubMed MeSH: Diabetes Mellitus, Type 2

Embase Emtree: type 2 diabetes mellitus

Free-text keywords (Title/Abstract):

type 2 diabetes; T2D; T2DM; NIDDM; noninsulin-dependent diabetes; non-insulin-dependent diabetes; "type II diabetes"

2. Exposure/measurement: continuous glucose monitoring

Controlled vocabulary (examples):

PubMed MeSH: Continuous Glucose Monitoring

Embase Emtree: continuous glucose monitoring

Free-text keywords (Title/Abstract):

continuous glucose monitoring; CGM; continuous glucose monitor; continuous glucose monitors; "real-time glucose monitoring"; "flash glucose monitoring"

3. Intervention: high-intensity interval training

Controlled vocabulary (examples):

PubMed MeSH: High-Intensity Interval Training

Embase Emtree: high intensity interval training

Free-text keywords (Title/Abstract):

high-intensity interval training; high intensity interval training; HIIT; HIIE; HIT; high-intensity intermittent exercise; sprint interval training; SIT; REHIT; low-volume HIIT; LVHIIT; HVHIIT; interval training; "aerobic interval training"; "interval exercise"  

4. Study design filter

To improve retrieval of eligible intervention studies, the following sensitive free-text terms were used:

randomized; randomised; random*; trial; controlled; intervention; clinical trial

Database-specific search strategies

| PubMed (MEDLINE)  Final search string (from inception to 26 Jan 2026; English): |
| --- |
| (("Diabetes Mellitus, Type 2"[Mesh] OR "type 2 diabetes"[Title/Abstract] OR T2D[Title/Abstract] OR T2DM[Title/Abstract] OR NIDDM[Title/Abstract] OR "noninsulin-dependent diabetes"[Title/Abstract] OR "non-insulin-dependent diabetes"[Title/Abstract]) AND  ("Continuous Glucose Monitoring"[Mesh] OR "continuous glucose monitoring"[Title/Abstract] OR CGM[Title/Abstract] OR "continuous glucose monitor"[Title/Abstract] OR "continuous glucose monitors"[Title/Abstract]) AND ("High-Intensity Interval Training"[Mesh] OR "high-intensity interval training"[Title/Abstract] OR "high intensity interval training"[Title/Abstract] OR HIIT[Title/Abstract] OR HIIE[Title/Abstract] OR HIT[Title/Abstract] OR "high-intensity intermittent exercise"[Title/Abstract] OR "sprint interval training"[Title/Abstract] OR SIT[Title/Abstract] OR REHIT[Title/Abstract] OR LVHIIT[Title/Abstract] OR HVHIIT[Title/Abstract] OR "interval training"[Title/Abstract]) AND (random*[Title/Abstract] OR trial[Title/Abstract] OR controlled[Title/Abstract] OR intervention*[Title/Abstract] OR "clinical trial"[Title/Abstract])) |
| Cochrane Library |
| ((MeSH descriptor: [Diabetes Mellitus, Type 2] explode all trees)  OR ("type 2 diabetes":ti,ab,kw OR T2D:ti,ab,kw OR T2DM:ti,ab,kw OR NIDDM:ti,ab,kw))  AND ((MeSH descriptor: [Continuous Glucose Monitoring] explode all trees) OR ("continuous glucose monitoring":ti,ab,kw OR CGM:ti,ab,kw)) AND ((MeSH descriptor: [High-Intensity Interval Training] explode all trees) OR ("high-intensity interval training":ti,ab,kw OR "high intensity interval training":ti,ab,kw OR HIIT:ti,ab,kw OR HIIE:ti,ab,kw OR HIT:ti,ab,kw OR "sprint interval training":ti,ab,kw OR SIT:ti,ab,kw OR REHIT:ti,ab,kw OR "interval training":ti,ab,kw)) AND (random*:ti,ab,kw OR trial:ti,ab,kw OR controlled:ti,ab,kw OR intervention*:ti,ab,kw) |
| Embase |
| ('type 2 diabetes mellitus'/exp OR 'type 2 diabetes':ti,ab OR t2d:ti,ab OR t2dm:ti,ab OR niddm:ti,ab OR 'noninsulin dependent diabetes':ti,ab OR 'non-insulin-dependent diabetes':ti,ab)  AND ('continuous glucose monitoring'/exp OR 'continuous glucose monitoring':ti,ab OR cgm:ti,ab) AND ('high intensity interval training'/exp OR 'high-intensity interval training':ti,ab OR 'high intensity interval training':ti,ab OR hiit:ti,ab OR hiie:ti,ab OR hit:ti,ab OR 'high-intensity intermittent exercise':ti,ab OR 'sprint interval training':ti,ab OR sit:ti,ab OR rehit:ti,ab OR lvhiit:ti,ab OR hvhiit:ti,ab OR 'interval training':ti,ab) AND (random*:ti,ab OR trial:ti,ab OR controlled:ti,ab OR intervention*:ti,ab OR 'clinical trial':ti,ab) |
| Web of Science Core Collection |
| TS=(("type 2 diabetes" OR T2D OR T2DM OR NIDDM OR "noninsulin-dependent diabetes" OR "non-insulin-dependent diabetes") AND ("continuous glucose monitoring" OR CGM OR "continuous glucose monitor" OR "continuous glucose monitors") AND ("high-intensity interval training" OR "high intensity interval training" OR HIIT OR HIIE OR HIT OR "high-intensity intermittent exercise" OR "sprint interval training" OR SIT OR REHIT OR LVHIIT OR HVHIIT OR "interval training") AND (random* OR trial OR controlled OR intervention* OR "clinical trial")) |
| SPORTDiscus (via EBSCOhost) |
| (("type 2 diabetes" OR T2D OR T2DM OR NIDDM OR "noninsulin-dependent diabetes" OR "non-insulin-dependent diabetes") AND ("continuous glucose monitoring" OR CGM OR "continuous glucose monitor" OR "continuous glucose monitors") AND ("high-intensity interval training" OR "high intensity interval training" OR HIIT OR HIIE OR HIT OR "high-intensity intermittent exercise" OR "sprint interval training" OR SIT OR REHIT OR LVHIIT OR HVHIIT OR "interval training") AND (random* OR trial OR controlled OR intervention* OR "clinical trial")) |

| Additional search |
| --- |
| Backward citation tracking: Reference lists of all included studies were manually screened to identify additional eligible trials not captured by the electronic searches. |
| Sensitivity search conducted during revision |
| During manuscript revision, additional sensitivity searches were conducted across all originally searched databases to assess the completeness of the original search strategy in response to peer-review comments. These supplementary searches incorporated reviewer-suggested terms that had not been explicitly listed in the original strategy, including “type II diabetes,” “real-time glucose monitoring,” “flash glucose monitoring,” “aerobic interval training,” and “interval exercise.”  In PubMed, Embase, Web of Science Core Collection, and SPORTDiscus, these sensitivity searches identified no records.  In the Cochrane Library, the sensitivity search identified 3 records, comprising 1 review article and 2 intervention studies not relevant to the present review question.  Therefore, no additional eligible studies were identified, and the study selection and overall conclusions of the review were unchanged. These sensitivity-search records were identified during manuscript revision and were kept separate from the original database yields reported above. |
| Data management |
| All retrieved records were exported to **EndNote X9** for reference management. Duplicate records were removed before title/abstract screening. The final original search was completed on **26 January 2026**. |
| Additional eligibility restrictions applied during screening |
| Conference abstracts, study protocols, trial registrations without published results, dissertations, theses, and other forms of grey literature were excluded. Only full-text, peer-reviewed original articles reporting interventional outcomes were considered eligible. |
| Database search results |
| The electronic searches yielded the following numbers of records before deduplication:  PubMed (MEDLINE): 24  Embase: 117  Web of Science Core Collection: 58  Cochrane Library: 239  SPORTDiscus (via EBSCOhost): 30 |
| After removal of duplicate records using EndNote X9, the remaining unique records were screened at the title and abstract level. |
