# Supplementary material for: High-intensity interval training and continuous glucose monitoring-derived glycemic outcomes in adults with type 2 diabetes: a systematic review and meta-analysis
Source: Front Endocrinol (Lausanne). 2026 Jun 17;17:1834479. doi: 10.3389/fendo.2026.1834479 (PMC13318697; doi:10.3389/fendo.2026.1834479)
Supplement: Supplementary file 4 [file DataSheet4.docx]

**Supplementary Table S2.**

Characteristics of continuous glucose monitoring protocols and reported CGM-derived outcomes in the included studies

| **Study** | **CGM device and monitoring details** | **Monitoring controls during CGM** | **Reported CGM-derived outcomes** |
| --- | --- | --- | --- |
| Little et al. (2011) | Medtronic CGMS iPro; interstitial glucose recorded/averaged every 5 min; capillary calibration 4 times/day; sensor placement not reported; 24-h CGM collected 48–72 h after the final training session | Medication unchanged; individualized control diet and diet replication; usual physical activity maintained | Mean 24-h glucose; postprandial glucose |
| Gillen et al. (2012) | Medtronic CGMS iPro (blinded); calibration 4 times/day; sensor placement not reported; 24-h post-exercise monitoring | Glucose-lowering medication unchanged; individualized diets; physical activity restricted and recorded | Mean 24-h glucose; postprandial glucose; time above range |
| Karstoft et al. (2013) | Guardian Real-Time with Sof-Sensor; calibration 3 times/day; sensor inserted in abdominal subcutaneous tissue; 3-day wear, with 2 full days analyzed | Medication monitored; 3-day diet record; accelerometer-based physical activity monitoring | Mean 24-h glucose; time above range; time below range |
| Karstoft et al. (2014) | Guardian Real-Time with Enlite sensor; 3-day continuous wear; sensor placement not reported; approximately 32 h of free-living glycemia assessed after MMTT | Antidiabetic medication paused; diet replicated/recorded; vigorous physical activity avoided and monitored | Mean 24-h glucose; time above range; time below range |
| Terada et al. (2016) | iPro2; calibration before meals and bedtime; sensor placement not reported; 24-h monitoring per condition | Medication standardized and withheld on testing mornings; breakfast standardized; physical activity restricted and steps recorded | Mean 24-h glucose; postprandial glucose; glycemic variability; time above range |
| Ruffino et al. (2017) | iPro; capillary calibration at regular intervals; sensor placed on the abdomen; approximately 20 h wear | Antidiabetic medication withheld; meals standardized; prior physical activity characterized | Mean 24-h glucose |
| Karstoft et al. (2017) | iPro2 with Enlite sensor; calibration 4 times/day; sensor inserted in abdominal subcutaneous adipose tissue; 24-h CGM aligned with urine collection | Glucose-lowering medication maintained; diet recorded/standardized; strenuous physical activity, alcohol, and caffeine restricted | Mean 24-h glucose; glycemic variability (MAGE); time above range; time below range |
| Metcalfe et al. (2018) | iPro2 with Enlite sensor; interstitial glucose recorded every 5 min; calibrated before meals and sleep; sensor placed on the abdomen; wear period from day 1 to day 3 | Medication maintained; standardized meals provided for approximately 42 h; physical activity restricted and monitored | Mean 24-h glucose; postprandial glucose; time above range |
| Winding et al. (2018) | Guardian Real-Time with Sof-Sensor; calibration 3 times/day; sensor inserted in abdominal subcutaneous tissue; 4-day wear, with 2 days analyzed | Medication unchanged; diet recorded; exercise avoided before testing | Mean 24-h glucose; glycemic variability (%CV); time above range; time below range; time in range |
| Savikj et al. (2019) | FreeStyle Libre; further technical details not explicitly reported; sensor placement not reported; 2-week baseline CGM and 2-week training-period CGM profiles | Medication maintained; diet recorded/replicated; exercise supervised, otherwise free-living | Mean 24-h glucose / 24-h glucose profiles |
| Marcotte-Chénard et al. (2021) | iPro 2 professional CGM with Enlite sensor; calibration 4 times/day; sensor inserted in the abdominal area; 4-day wear with control, exercise, and post-exercise days analyzed | Medication timing recorded; meals/snacks recorded; unusual physical activity restricted | Mean 24-h glucose; peak glucose; mild hyperglycemia; hyperglycemia; hypoglycemia; glycemic variability (SD, CONGA1) |
| Marcotte-Chénard et al. (2023) | Dexcom G6; 2-h warm-up followed by calibration; sensor inserted in the abdominal area; 10-day wear, with each condition analyzed over 24 h | Medication maintained/recorded; standardized breakfast and lunch; dietary intake replicated; strenuous physical activity restricted | Mean 24-h glucose; peak glucose; moderate hyperglycemia; hyperglycemia; hypoglycemia; glycemic variability (SD, CONGA1, MAGE) |

Abbreviations: CGM, continuous glucose monitoring; MAGE, mean amplitude of glycemic excursions; CONGA1, continuous overall net glycemic action over 1 h; SD, standard deviation; %CV, coefficient of variation; MMTT, mixed-meal tolerance test. “Not reported” indicates that the information was not explicitly described in the original article.
