# Supplementary material for: High-intensity interval training and continuous glucose monitoring-derived glycemic outcomes in adults with type 2 diabetes: a systematic review and meta-analysis
Source: Front Endocrinol (Lausanne). 2026 Jun 17;17:1834479. doi: 10.3389/fendo.2026.1834479 (PMC13318697; doi:10.3389/fendo.2026.1834479)
Supplement: Supplementary file 5 [file DataSheet5.docx]

Supplementary Table S3. Characteristics of HIIT and comparator exercise protocols in the included studies

| Study | Group | Mode | Intensity prescription | Interval structure / protocol | Session duration | Frequency | Intervention duration | Adaptation / progression |
| --- | --- | --- | --- | --- | --- | --- | --- | --- |
| Little et al. (2011) | HIIT | Cycle ergometer | ~90% HRmax | Warm-up 3 min; 10 × (1 min work / 1 min recovery); cool-down 2 min | ~25 min | 3/week | 2 weeks | 1–2 familiarization sessions |
| Gillen et al. (2012) | HIIT | Cycle ergometer | 85 ± 7% HRmax | Warm-up 3 min; 10 × (1/1 min); cool-down 2 min | ~25 min | 1 session | Acute | 1–2 familiarization sessions |
| Karstoft et al. (2013) | HIIT | Treadmill walking | ~85% VO₂peak | 10 × (3 min work / 3 min recovery at ~40% VO₂peak) | ~60 min | 5/week | 16 weeks | None reported |
|  | MICT | Treadmill walking | ~55% VO₂peak | Continuous | ~60 min | 5/week | 16 weeks | — |
| Karstoft et al. (2014) | HIIT | Treadmill walking | ~90% VO₂peak | 10 × (3 min work / 3 min recovery at ~55% VO₂peak) | NR | NR | NR | 1–2 weeks |
|  | MICT | Treadmill walking | ~70% VO₂peak | Continuous | ~60 min | NR | NR | — |
| Terada et al. (2016) | HIIT | Treadmill walking | 100% VO₂peak | 15 × (1 min work / 3 min recovery at ~40% VO₂peak) | ~60 min | 1 session | Acute | Progressive increase in session duration over weeks 1–3 |
|  | MICT | Treadmill walking | ~55% VO₂peak | Continuous | ~60 min | 1 session | Acute | — |
| Ruffino et al. (2017) | HIIT | Cycle ergometer | All-out sprint | Progressive REHIT/SIT-type sprint protocol | ~6 min 25 s | 3/week | 8 weeks | 1-week progression |
|  | MICT | Walking | 40–55% HRR | Continuous | ~30 min | 5/week | 8 weeks | Progressive intensity by week |
| Karstoft et al. (2017) | HIIT | Treadmill walking | ~89% VO₂peak | 10 × (3 min work / 3 min recovery at ~54% VO₂peak) | NR | 5/week | 2 weeks | None reported |
|  | MICT | Treadmill walking | ~73% VO₂peak | Continuous | ~60 min | 5/week | 2 weeks | — |
| Winding et al. (2018) | HIIT | Cycle ergometer | 95% Wpeak | Warm-up 5 min; 10 × (1 min work / 1 min recovery at 20% Wpeak) | NR | 3/week | 11 weeks | None reported |
|  | MICT | Cycle ergometer | 50% Wpeak | Continuous | ~40 min | 3/week | 11 weeks | — |
| Metcalfe et al. (2018) | HIIT | Cycle ergometer | All-out sprint | 2 × 20 s all-out | NR | NR | NR | None reported |
|  | REHIT | Cycle ergometer | ~90% HRmax | 10 × 1 min at ~90% HRmax | NR | NR | NR | — |
|  | MICT | Cycle ergometer | 50% Wpeak | Warm-up 2 min; continuous; cool-down 2 min | ~30 min | NR | NR | — |
| Savikj et al. (2019) | HIIT | Cycle ergometer | Workload NR | Warm-up 7 min; 6 × (1 min work / 1 min recovery) | NR | NR | NR | None reported |
| Chénard et al. (2021) | HIIT | Treadmill walking | 90% HRR | Warm-up 3 min; 6 × (1 min work / 2 min recovery at 45% HRR); cool-down 2 min | NR | 3/week | 12 weeks | 4-week adaptation period |
|  | MICT | Treadmill walking | 60% HRR | Warm-up 2 min; continuous; cool-down 3 min | ~45 min | 3/week | 12 weeks | — |
| Chénard et al. (2023) | HIIT4 | Treadmill walking | 90% HRmax | Warm-up 5 min; 4 × 4 min with 3-min recovery at 70% HRmax; cool-down 2 min | ~35 min | 1 session | Acute | 4–5 familiarization sessions |
|  | HIIT1 | Treadmill walking | 90% HRmax | Warm-up 10 min; 10 × (1 min work / 1 min recovery at 70% HRmax); cool-down 8 min | ~38 min | 1 session | Acute | — |

Abbreviations: HIIT, high-intensity interval training; MICT, moderate-intensity continuous training; REHIT, reduced-exertion high-intensity interval training; SIT, sprint interval training; HRmax, maximal heart rate; HRR, heart rate reserve; VO₂peak, peak oxygen uptake; Wpeak, peak power output; NR, not reported.
