# Supplementary material for: High-intensity interval training and continuous glucose monitoring-derived glycemic outcomes in adults with type 2 diabetes: a systematic review and meta-analysis
Source: Front Endocrinol (Lausanne). 2026 Jun 17;17:1834479. doi: 10.3389/fendo.2026.1834479 (PMC13318697; doi:10.3389/fendo.2026.1834479)
Supplement: Supplementary file 7 [file DataSheet7.docx]

Supplementary Table S5

a. Subgroup analyses for the effect of HIIT versus non-exercise control on mean 24-h glucose

| Subgroup | Comparisons, n | Effect estimate (MD, 95% CI) | p value | Heterogeneity |
| --- | --- | --- | --- | --- |
| Overall | 14 | −0.51 (−0.96 to −0.06) | 0.03 | Chi² = 82.07, p < 0.001; I² = 84% |
| BMI |  |  |  |  |
| >25 kg/m² | 8 | −0.43 (−1.02 to 0.16) | 0.15 | Chi² = 79.25, p < 0.001; I² = 91% |
| >30 kg/m² | 6 | −0.67 (−1.17 to −0.17) | 0.009 | Chi² = 2.29, p = 0.81; I² = 0% |
| Subgroup difference | — | — | < 0.001 | — |
| Intervention duration |  |  |  |  |
| >2 weeks | 2 | −1.64 (−2.88 to −0.4) | 0.009 | Chi² = 1.10, p = 0.29; I² = 9% |
| ≤2 weeks | 12 | −0.41 (−0.88 to 0.05) | 0.08 | Chi² = 75.62, p < 0.001; I² = 85% |
| Subgroup difference | — | — | < 0.001 | — |
| CGM monitoring window |  |  |  |  |
| ≥48 h | 2 | −1.64 (−2.88 to −0.4) | 0.009 | Chi² = 1.10, p = 0.29; I² = 9% |
| <48 h | 12 | −0.41 (−0.88 to 0.05) | 0.08 | Chi² = 75.62, p < 0.001; I² = 85% |
| Subgroup difference | — | — | < 0.001 | — |

Supplementary Table S5b. Subgroup analyses for the effect of HIIT versus MICT on mean 24-h glucose

| Subgroup | Comparisons, n | Effect estimate (MD, 95% CI) | p value | Heterogeneity |
| --- | --- | --- | --- | --- |
| Overall | 11 | −0.20 (−0.36 to −0.04) | 0.01 | Chi² = 7.69, p = 0.66; I² = 0% |
| BMI |  |  |  |  |
| >25 kg/m² | 6 | −0.26 (−0.45 to −0.08) | 0.01 | Chi² = 4.77, p = 0.45; I² = 0% |
| >30 kg/m² | 5 | −0.03 (−0.33 to 0.27) | 0.85 | Chi² = 1.22, p = 0.87; I² = 0% |
| Subgroup difference | — | — | 0.198 | — |
| Intervention duration |  |  |  |  |
| >2 weeks | 3 | −0.15 (−0.43 to 0.12) | 0.27 | Chi² = 5.58, p = 0.23; I² = 28% |
| ≤2 weeks | 8 | −0.22 (−0.41 to −0.03) | 0.02 | Chi² = 1.96, p = 0.85; I² = 0% |
| Subgroup difference | — | — | 0.678 | — |
| CGM monitoring window |  |  |  |  |
| ≥48 h | 2 | −0.16 (−0.32 to 0.00) | 0.06 | Chi² = 3.67, p = 0.89; I² = 0% |
| <48 h | 9 | −0.68 (−1.24 to −0.12) | 0.02 | Chi² = 0.99, p = 0.32; I² = 0% |
| Subgroup difference | — | — | 0.08 | — |

Note: Effect estimates are presented as mean differences (MDs) with 95% confidence intervals (95% CIs). HIIT, high-intensity interval training; MICT, moderate-intensity continuous training; CGM, continuous glucose monitoring. “Comparisons, n” refers to the number of comparison units included in each subgroup analysis rather than the number of independent studies.

The full subgroup analysis results for mean 24-h glucose are presented in **Supplementary Tables S5a and S5b.**
